# Genetic Disruption of KLF1 K74 SUMOylation in Hematopoietic System Promotes Healthy Longevity in Mice

**DOI:** 10.1002/advs.202201409

**Published:** 2022-07-13

**Authors:** Yu‐Chiau Shyu, Po‐Cheng Liao, Ting‐Shou Huang, Chun‐Ju Yang, Mu‐Jie Lu, Shih‐Ming Huang, Xin‐Yu Lin, Cai‐Cin Liou, Yu‐Hsiang Kao, Chi‐Huan Lu, Hui‐Ling Peng, Jim‐Ray Chen, Wen‐Jin Cherng, Ning‐I Yang, Yung‐Chang Chen, Heng‐Chih Pan, Si‐Tse Jiang, Chih‐Chin Hsu, Gigin Lin, Shin‐Sheng Yuan, Paul Wei‐Che Hsu, Kou‐Juey Wu, Tung‐Liang Lee, Che‐Kun James Shen

**Affiliations:** ^1^ Community Medicine Research Center Chang Gung Memorial Hospital Keelung branch Keelung 204 Taiwan; ^2^ Department of Nursing Chang Gung University of Science and Technology Taoyuan 333 Taiwan; ^3^ Department of General Surgery Chang Gung Memorial Hospital Keelung branch Keelung 204 Taiwan; ^4^ School of Traditional Chinese Medicine College of Medicine Chang Gung University Taoyuan 333 Taiwan; ^5^ Department of Radiation Oncology Chung‐Gung Memorial Hospital Keelung branch Keelung 204 Taiwan; ^6^ Department of Pathology Chang Gung Memorial Hospital Keelung branch Keelung 204 Taiwan; ^7^ Department of Cardiology Chang Gung Memorial Hospital Linkou branch Taoyuan 333 Taiwan; ^8^ Department of Cardiology Chang Gung Memorial Hospital Keelung branch Keelung 204 Taiwan; ^9^ Department of Nephrology Chang Gung Memorial Hospital Linkou branch Taoyuan 333 Taiwan; ^10^ Department of Medicine School of Medicine Chang Gung University Taoyuan 333 Taiwan; ^11^ Department of Research and Development National Laboratory Animal Center Tainan 741 Taiwan; ^12^ Department of Physical Medicine and Rehabilitation Chang Gung Memorial Hospital Keelung branch Keelung 204 Taiwan; ^13^ Department of Medical Imaging and Intervention Chang Gung Memorial Hospital Linkou branch Taoyuan 333 Taiwan; ^14^ Clinical Metabolomics Core Lab Chang Gung Memorial Hospital Linkou branch Taoyuan 333 Taiwan; ^15^ Department of Medical Imaging and Radiological Sciences Chang Gung University Taoyuan 333 Taiwan; ^16^ Institute of Statistical Science Academia Sinica Taipei 115 Taiwan; ^17^ Institute of Molecular and Genomic Medicine National Health Research Institute Zhunan 350 Taiwan; ^18^ Cancer Genome Research Center Chang Gung Memorial Hospital Linkou branch Taoyuan 333 Taiwan; ^19^ Pro‐Clintech Co. Ltd. Keelung 204 Taiwan; ^20^ Institute of Molecular Biology Academia Sinica Taipei 115 Taiwan; ^21^ Ph.D. Program in Medical Neuroscience Taipei Medical University Taipei 110 Taiwan

**Keywords:** aging, antiaging, hematopoietic stem cells (HSCs), KLF1/EKLF, SUMOylation

## Abstract

The quest for rejuvenation and prolonged lifespan through transfusion of young blood has been studied for decades with the hope of unlocking the mystery of the key substance(s) that exists in the circulating blood of juvenile organisms. However, a pivotal mediator has yet been identified. Here, atypical findings are presented that are observed in a knockin mouse model carrying a lysine to arginine substitution at residue 74 of Krüppel‐like factor 1 (KLF1/EKLF), the SUMOylation‐deficient *Klf1* ^K74R/K74R^ mouse, that displayed significant improvement in geriatric disorders and lifespan extension. *Klf1* ^K74R/K74R^ mice exhibit a marked delay in age‐related physical performance decline and disease progression as evidenced by physiological and pathological examinations. Furthermore, the KLF1(K74R) knockin affects a subset of lymphoid lineage cells; the abundance of tumor infiltrating effector CD8^+^ T cells and NKT cells is increased resulting in antitumor immune enhancement in response to tumor cell administration. Significantly, infusion of hematopoietic stem cells (HSCs) from *Klf1* ^K74R/K74R^ mice extends the lifespan of the wild‐type mice. The *Klf1* ^K74R/K74R^ mice appear to be an ideal animal model system for further understanding of the molecular/cellular basis of aging and development of new strategies for antiaging and prevention/treatment of age‐related diseases thus extending the healthspan as well as lifespan.

## Graphical abstract

The quest for prolonged lifespan has been ongoing for decades with the hope of unlocking the mystery of the key substance(s) in longevity. This study presents a longevity mouse model that significantly improves geriatric disorders and lifespan in a hematopoietic manner. This animal model is invaluable for exploring new approaches for antiaging and offers a contribution to advancing geroscience.

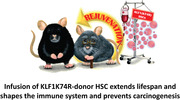

## 1 Introduction

Aging is a progressive and irreversible degeneration process affected by endogenous genetic factors and exogenous environmental factors.^[^ ^1^ ^]^ It is associated with the functional decline of all tissues and a striking increase in many diseases, such as cardiovascular (CV) disease, diabetes, cancer, arthritis, and frailty.^[^ ^1^, ^2^ ^]^ Although aging has long been considered an irreversible one‐way street, rejuvenation strategies have recently been employed that target various hallmarks of aging, including systemic factors in the blood, metabolic manipulation, senescent cell ablation, and cellular reprogramming.^[^ ^3^ ^]^

Transfusion of young blood has been proposed as a possible approach for improved age‐related impairments, which are contributed mainly by blood factors rather than blood cells.^[^ ^4^ ^]^ Studies of heterochronic parabiosis and plasma transfer identified several circulating factors that have recently been considered as targets for rejuvenation, such as C‐C Motif Chemokine Ligand 11 (CCL11), *β*2‐microglobulin, oxytocin, TIMP Metallopeptidase Inhibitor 2 (TIMP2), and Growth/differentiation factor 11 (GDF11), that negatively or positively influence aspects of aging progression.^[^ ^4^, ^5^ ^]^ Furthermore, metabolic manipulation through long‐term dietary restriction (DR) and calorie restriction (CR) have been found to extend healthspan and lifespan by modulating the activity of the 5'AMP‐activated protein kinase (AMPK)/mammalian target of rapamycin (mTOR) and insulin/insulin‐like growth factor (IGF) pathway which plays a pivotal role in nutrient sensing events.^[^ ^6^ ^]^ Both genetic manipulations and nongenetic approaches have been used on animals to understand and modify known age‐related complex and intercrossing pathways, including growth hormone signaling,^[^ ^7^ ^]^ and pathways involving the target of rapamycin (TOR),^[^ ^8^ ^]^ nutritional signaling, mitochondrial biogenesis,^[^ ^9^ ^]^ energy metabolism,^[^ ^10^ ^]^ thermogenesis,^[^ ^11^ ^]^ inflammation,^[^ ^12^ ^]^ and insulin signaling.^[^ ^1a^ ^]^ More recently, ablation of senescent cells by genetic manipulation or senolytic agents that target p16‐positive cells or inhibition of the B‐cell lymphoma 2 (Bcl‐2) protein family, navitoclax,^[^ ^13^ ^]^ tyrosine kinases,^[^ ^14^ ^]^ p53‐Murine double minute 2 (MDM2) interaction,^[^ ^15^ ^]^ and heat shock protein 90 (Hsp90)^[^ ^16^ ^]^ have shown that reduced senescent cell accumulation can ameliorate or retarding age‐related dysfunction in tissues.

The establishment and research of different animal models is essential to understanding the regulatory mechanisms of the genetics of aging and healthspan. Here, we report the generation of a healthy, long‐living *Klf1* ^K74R/K74R^ knockin mouse model that abolished the small ubiquitin‐like modifier (SUMO) acceptor site, through gene targeting of the Krüppel‐Like Factor 1 (*Klf1*; *Eklf*: Erythroid Krüppel‐Like Factor) gene, a conserved zinc finger‐containing DNA‐binding transcription factor initially identified in erythroid lineages and recognized for its functions in erythropoiesis.^[^ ^17^ ^]^ Later studies revealed additional functions of KLF1 in biomolecular pathways, such as common myeloid progenitor differentiation, cell fate decision in megakaryocyte‐erythrocyte bipotential progenitors, erythrocyte membrane stability, maintenance of erythroblastic islands, and cell cycle regulation.^[^ ^18^ ^]^ Notably, SUMOylation of mouse KLF1 protein at lysine 74 (K74), a conserved SUMOylation site among vertebrate KLF1 proteins, modulates its transcriptional regulatory property and/or nuclear import during erythroid differentiation.^[^ ^19^ ^]^ Further elucidation of the in vivo role of SUMOylated KLF1 awaits the generation of mice with SUMOylation‐deficient KLF1 mutant. In this study, we developed a *Klf1* ^K74R/K74R^ mouse model that displayed intriguing unanticipated frailty reduction and lifespan extension phenotypes. *Klf1* ^K74R/K74R^ mice exhibited normal behavioral and physiological features that were associated with better physical performance, less aging dependent organ fibrosis, neuronal function decay during aging, and an exceedingly low cancer incidence in older adult animals. Allogeneic donor lymphocyte infusion or repeated infusion of HSCs by employed *Klf1* ^K74R/K74R^ mice donor cells displayed similar promising results in attenuating tumor growth and impact on lifespan in mice. Therefore, here we present *Klf1* ^K74R/K74R^ mice as an ideal model system for both basic and translational research to study the prevention and treatment of aging‐related diseases for extending the lifespan and healthspan of animals.

## 2 Results

### 2.1 KLF1(K74R) Knockin Increases the Lifespan of Klf^1K74R/K74R^ Mice

**Figure 1:**
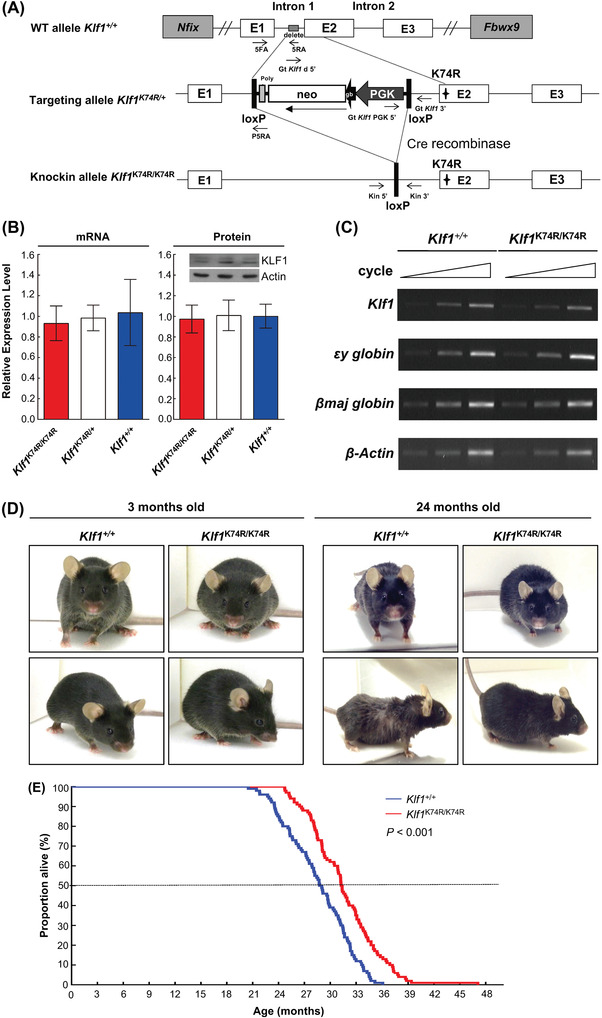
Genetically engineered *Klf1* ^K74R/K74R^ mice exhibit increased lifespan. A) A map of the loxP‐PGK‐gb2‐neo‐loxP K74R retroviral vector. The protein‐encoding portion of exon 2 of the *Klf1* gene was replaced (asterisks) by loxP‐PGK‐gb2‐neo‐loxP K74R retroviral vector and a neomycin cassette (Neo, open box) flanked by lox P sites (black box). After homologous recombination, the neomycin cassette was excised by Cre‐mediated recombination. B) *Klf1* mRNA expression was analyzed by quantitative RT‐PCR. Total RNA was extracted from bone marrow samples using TRIzol reagent (*n* = 3, left panel). Data are presented as mean ± SD. Expression of KLF1 protein was quantified by western blots in E13.5 fetal liver (*n* = 3, right panel). C) Semi‐quantitative RT‐PCR analysis of *Klf1* and downstream genes. Total RNA samples were isolated from E13.5 fetal livers. D) Appearance of young and old *Klf1* ^K74R/K74R^ and *Klf1* ^+/+^mice. E) Kaplan–Meier survival curves of the *Klf1* ^K74R/K74R^ and *Klf1* ^+/+^ mice (*n* = 100 mice for each genotype). Each data point represents one animal. Statistical significance was assessed using the original method of the log‐rank test.

A previous study showed that SUMOylation of KLF1 does not influence the transcriptional activity of the N‐terminal transactivation domain.^[^ ^20^ ^]^ We further attempted to characterize whether SUMOylation at K74 affects DNA binding activity of KLF1. To assess the DNA binding capability of SUMOylated KLF1, we constructed and expressed a recombinant SUMO‐KLF1△73 fusion by directly fusing SUMO‐1 with an N‐terminal Lysine residue of truncated KLF1 (aa 74–376) to mimic SUMOylated KLF1 and evaluated the effect by electrophoretic mobility shift assay (EMSA) (Figure S1A, Supporting Information). The results showed that the CACCC box binding affinity of Flag‐SUMO‐KLF1△73 was stronger than either Flag‐KLF1 or Flag‐KLF1△73 (compare lane 5 to lanes 3 and 4 in Figure S1A, left panel, Supporting Information), the migration band of these variable KLF1/DNA complexes was also confirmed by anti‐Flag antibody supershift analysis (lanes 6 to 9 in Figure S1A, left panel, Supporting Information). In addition, the comparable binding affinity between Flag‐KLF1 and Flag‐KLF1△73 (lanes 3 and 4 in Figure S1A, left panel, Supporting Information) suggests that the enhancement of DNA binding capability of Flag‐SUMO‐KLF1△73 protein occurred in a SUMOylation dependent manner rather than the truncation of N‐terminal domain. For a thorough understanding of the in vivo role of KLF1 SUMOylation, we generated knockin mice carrying homozygous K74R substitution (conversion of K74 to arginine (R) in KLF1 by conventional gene targeting that abolished the SUMOylation of KLF1 (**Figure** **1**A and Figure S1B, Supporting Information). The presence of the K74R mutation of *Klf1* gene was confirmed by PCR genotyping and Sanger sequencing in heterozygous *Klf1* ^K74R/+^ and homozygous *Klf1* ^K74R/K74R^ mice, respectively (Figure S1C, D, Supporting Information). Genotypic and molecular analyses revealed *Klf1* ^K74R/K74R^ mice produced offspring inheriting the K74R mutation at the expected Mendelian ratio, and the expression of *Klf1* mRNA and protein were not altered by this K74R mutation in either heterozygous *Klf1* ^K74R/+^ or homozygous *Klf1* ^K74R/K74R^ mice that were comparable to their wild‐type (*Klf1* ^+/+^) littermates (Figure 1B). Unlike the embryonic lethality of KLF1 knockout mice,^[^ ^20^ ^]^ *Klf1* ^K74R/K74R^ mice did not display any clear abnormality in globin synthesis. Gene expression analysis revealed that the expression of KLF1 downstream target *β*‐globin genes, *εy*‐ and *β*_maj_‐globin, were not significantly affected in *Klf1* ^K74R/K74R^ mice compared to *Klf1* ^+/+^ mice (Figure 1C). The genes nearby *Klf1*, i.e., *Gcdh*, *Dnase2a*, *Mast1* and *Syce2*, were similar between *Klf1* ^K74R/K74R^ and *Klf1* ^+/+^ mice, respectively (Figure S1E, Supporting Information). Surprisingly, *Klf1* ^K74R/K74R^ mice were healthy and displayed regular ambulatory activity without any visible defects over an extended period of observation for any disorders that may develop in adulthood or later life. Intriguingly, despite there being no significant pathological events identified in *Klf1* ^K74R/K74R^ mice upon abolishment of SUMOylation of KLF1, we found completely different geriatric appearance and behavior among elderly *Klf1* ^K74R/K74R^ mice and *Klf1* ^+/+^ mice. The elderly *Klf1* ^K74R/K74R^ mice did not show any aging features, such as hunched posture, sparse or discolored hair coats, or ulcerated or bleeding tumors (Figure 1D). Encouraged by these findings, we proceeded to conduct a survival study by establishing large cohorts of *Klf1* ^K74R/K74R^ and *Klf1^+/+^* mice littermates that were maintained in a barrier facility for age‐related decline determination and allowed them to die spontaneously. Initially, we determined the lifespan of these cohorts. The observed survival curves revealed a statistically significant lifespan extension in both sexes of the *Klf1* ^K74R/K74R^ cohort compared to the *Klf1* ^+/+^ counterpart (*p* < 0.001, Figure 1E and Figure S1F,G, Supporting Information). The median lifespan of *Klf1* ^K74R/K74R^ mice (30.75 months) was 2.45 months longer than that of the wild‐type counterparts (28.3 months) (*p* < 0.001, **Table** **1**). Next, we determined the minimum and maximum survival of each cohort by evaluating the average lifespan of the youngest and oldest 10% of mice within a cohort, respectively.^[^ ^21^ ^]^ The mean lifespan of the youngest and oldest 10% of the cohort of *Klf1* ^K74R/K74R^ mice (38.07 and 25.01 months) were 4.19 and 2.92 months longer than in *Klf1* ^+/+^ mice, respectively. which was longer than those of previously reported longevity mouse models, such as CDGSH iron–sulfur domain‐containing protein 2 (*Cisd2*) TG mice^[^ ^10^ ^]^ and *Myc* haploinsufficient (*Myc* ^+/−^) mice.^[^ ^22^ ^]^ The median lifespan of another C57BL/6J line of mice, line #86 *Klf1* ^K74R/K74R^ (30.85 months) was 2.55 months longer than that of the wild‐type counterparts (*p* < 0.05, Figure S1H). Moreover, the lifespan of longest living *Klf1* ^K74R/K74R^ mouse was reached 46.33 months (Table 1), Together, this analysis implies that KLF1(K74R) knockin has an intrinsic impact on the delay of early mortality and the extension of lifespan under normal housing conditions.

**Table 1:** Comparative survival characteristics of *Klf1* ^+/+^ and *Klf1* ^K74R/K74R^ mice

		Lifespan (months)						
Group	Genotype	Median	Mean	Oldest 10%	Youngest 10%	Maximum	Minimum	*n*
Male	*Klf1* ^K74R/K74R^	31.18	31.39 ± 4.13	38.66 ± 3.08	24.80 ± 0.41	46.33	24.30	77
	*Klf1* ^+/+^	29.08	28.73 ± 3.59	34.14 ± 0.63	22.48 ± 1.08	35.48	20.16	77
Female	*Klf1* ^K74R/K74R^	29.11	29.97 ± 2.81	35.75 ± 0.97	35.75 ± 0.17	36.59	25.70	23
	*Klf1* ^+/+^	24.89	26.48 ± 3.53	32.44 ± 0.74	21.26 ± 0.21	33.08	21.08	23
**Total**								
	*Klf1* ^K74R/K74R^	30.75	31.07 ± 3.90	38.07 ± 2.78	25.01 ± 0.57	46.33	24.30	100
	*Klf1* ^+/+^	28.30	28.21 ± 3.69	33.88 ± 0.69	22.09 ± 0.97	35.48	20.16	100

### 2.2 KLF1(K74R) Knockin Improves Age‐Related Physiological and Physical Changes in Mice

**Figure 2:**
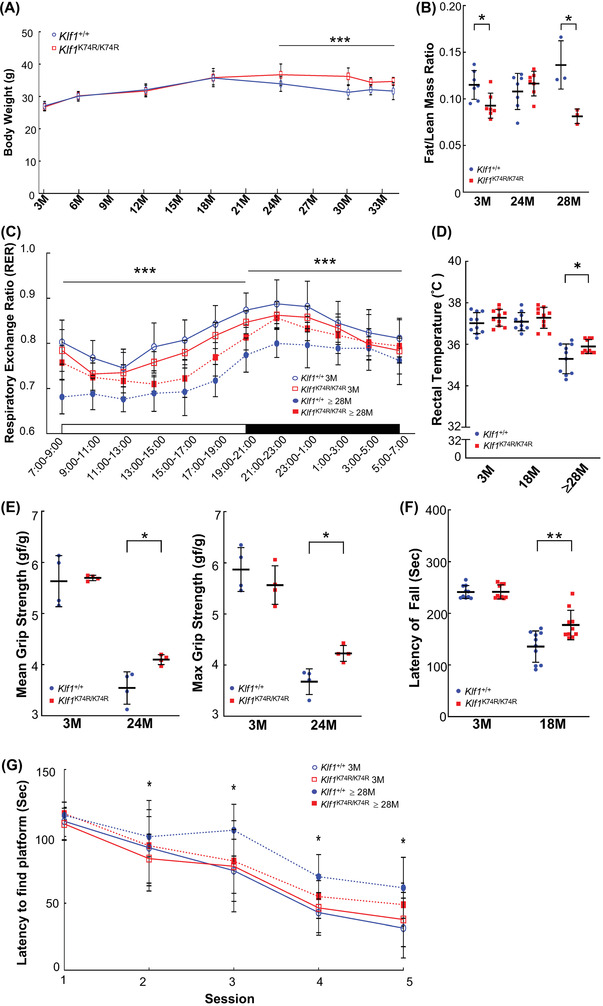
Genetically engineered *Klf1* ^K74R/K74R^ mice exhibit increased healthspan. A) Body weights were recorded at ages 3, 6, 12, 18, 24, 30, 32, and ≥ 34 months; the number of mice in each group was 30, 30, 30, 30, 30, 30, 8, and 6, respectively. B) Fat/lean mass ratio of *Klf1* ^K74R/K74R^ mice were compared with *Klf1* ^+/+^ mice at age 3, 24, and 28 months. The numbers of mice in each group were 7, 7, and 4, respectively. C) RER examination; the canonical diurnal metabolic parameters were measured in young (3 months old) and old (≥ 28 months old) *Klf1* ^K74R/K74R^ and *Klf1* ^+/+^ mice (*n* = 6/group). D) Rectal temperatures were recorded at ages of 3, 18, and above 28 months (*n* = 10/group). E) Grip strength of *Klf1* ^K74R/K74R^ and *Klf1* ^+/+^ mice was recorded at age 3 and 24 months (*n* = 4/group). F) Rotarod performance test. The latencies to fall from an accelerating rotarod were compared between *Klf1* ^K74R/K74R^ and *Klf1* ^+/+^ mice at age 3 and 18 months, respectively (*n* = 10/group). G) Morris water maze test. The latencies of finding the platform underwater in different sections of training were compared between groups. All the data were assessed by ANCOVA or Student's *t*‐test and presented as mean ± SD and *p ≤ 0.05, **p ≤ 0.01, ***p ≤ 0.001.

To evaluate the phenotypic effects and functional consequence of KLF1(K74R) mutation as associated with underlying causes of lifespan extension phenotype in *Klf1* ^K74R/K74R^ mice, we performed a longitudinal examination of body composition parameters and certain physical and metabolic aspects associated with age‐related decline. Among these examinations, we noticed the trend in mean body weight change by age was significantly different in *Klf1* ^K74R/K74R^ and *Klf1* ^+/+^ mice (*p* < 0.001, **Figure** **2**A). A normal age‐dependent progressive body weight loss of *Klf1^+/+^* mice was observed from ages 18 to 30 months (35.69 ± 2.13 g to 31.31 ± 2.09 g), in contrast, the *Klf1* ^K74R/K74R^ mice were resistant to age‐related body weight loss (35.84 ± 2.76 g to 36.20 ± 2.62 g) (Figure 2A). Evaluation of body composition by NMR measurement showed that aged *Klf1* ^K74R/K74R^ mice can maintain their lean mass in later life (*Klf1* ^K74R/K74R^, 76.48 ± 1.39% to 76.53 ± 0.47% and *Klf1* ^+/+^, 75.76 ± 0.90% to 73.95 ± 0.65%), while resistance to aged‐related fat mass increases (8.84 ± 0.85% to 6.22 ± 0.50%) in comparison with *Klf1* ^+/+^ mice (8.16 ± 1.31% to 10.07 ± 1.50%) at age 24 to 28 months (Table S1, Supporting Information). Consistent with the alteration in age‐related body weight changes, the fat to lean mass (F/L) ratio was still well maintained in aged *Klf1* ^K74R/K74R^ mice, which was relatively lower than that in *Klf1* ^+/+^ mice (Figure 2B). Likewise, the improvement in age‐related decline on basal metabolic rate, which is relevant to alterations in body weight and body composition, was also observed in aged *Klf1* ^K74R/K74R^ mice. Indirect calorimetry (IC) analysis showed that aged *Klf1* ^K74R/K74R^ mice had a significantly higher respiratory exchange ratio (RER) value (night cycle, 0.83 ± 0.04 to 0.82 ± 0.04, and day cycle 0.78 ± 0.05 to 0.74 ± 0.05) than aged *Klf1* ^+/+^ mice (night cycle, 0.85 ± 0.05 to 0.79 ± 0.04 and day cycle 0.80 ± 0.06 to 0.70 ± 0.05), that was nearly comparable with the RER of night cycle in the young *Klf1* ^K74R/K74R^ mouse group (Figure 2C and Figure S2A–C, Supporting Information). Notably, this effect was not associated with the energy intake, and the food uptake and water consumption showed no significant difference between *Klf1* ^K74R/K74R^ and *Klf1* ^+/+^ mice in either the young or aged groups being fed normal chow (*p* < 0.05, Figure S2D,E). A similar effect was also observed in the aging‐associated core temperature (*T*_co_) decrease^[^ ^23^ ^]^ between *Klf1* ^K74R/K74R^ and *Klf1* ^+/+^ mice. The T_co_ was assessed by rectal temperature measurement at ages 3 months, 18 months, and older than 28 months, respectively. The results showed that the mean T_co_ was significantly reduced in both *Klf1* ^+/+^ or *Klf1* ^K74R/K74R^ mice at age over 28 months compared with age 18 months (4.83%, *p* < 0.005 and 3.73%, *p* < 0.005), notably, (Figure 2D). Next, we evaluated whether the *Klf1* ^K74R/K74R^ mice showed an improvement in the impairments in physical performance and mental integrity that exist in elderly mice. To assess the impact of locomotor performance during the progression of aging, muscle strength and motor coordination were examined by handgrip strength and rotarod test, respectively. There were no significant differences in the grip strengths and motor coordination among the young *Klf1* ^+/+^ and *Klf1* ^K74R/K74R^ mice at age 3 months (Figure 2E). Consistently, a significant retardation in locomotor ability decline due to advanced age was observed in aged *Klf1* ^K74R/K74R^ mice, which was examined by rotarod test which showed greater endurance compared with *Klf1* ^+/+^ mice at the corresponding age (Figure 2F). Furthermore, a small‐scale Morris water maze test revealed that *Klf1* ^K74R/K74R^ mice exhibited better spatial learning and memory capability than *Klf1* ^+/+^ mice in old age groups (age over 28 months) (Figure 2G). These data suggest that *Klf1* ^K74R/K74R^ mice are resistant to age‐related decline in metabolic and physical function and further support the potential impact of KLF1(K74R) knockin on the modulation of age‐related physiological declines.

### 2.3 KLF1(K74R) Knockin Delays the Onset of Age‐Related Disorders

To expand on these findings, we further assessed whether KLF1(K74R) knockin affects the progression of geriatric disorders and organ function deterioration with advancing age by carrying out a longitudinal analysis on dyslipidemia, chronic hepatic disorders, diabetes mellitus, hypertension, CV disorders, chronic nephropathy, and neoplasia, which are associated with increased risk of morbidity and mortality in aged mice. For biochemical evaluation of the effects of KLF1(K74R) knockin, peripheral blood serum analysis was performed, and the results were compared across genotypes and age groups. The lipid metabolism changes were characterized by the levels of serum lipids, such as total cholesterol (TCHO), triglyceride (TG), and high‐density lipoprotein (HDL). Blood concentrations of fasting glucose and Insulin‐Like Growth Factor‐1 (IGF‐1) as well as glucose tolerance were also examined. The results showed no significant changes in *Klf1* ^K74R/K74R^ mice compared to their age matched *Klf1^+/+^* mice (Figure S2F–J). The measurements of systolic blood pressure (SBP) and diastolic blood pressure (DBP) in different age groups showed that the onset of hypertension or pre‐hypertension with advancing age was not yet observed in among the *Klf1^+/+^* and *Klf1* ^K74R/K74^ *^R^* mouse groups (Figure S2K,L). Notably, the resting heart rate (RHR) analysis showed that the average RHR of the aged group of *Klf1* ^+/+^ mice was significantly lower (11.6%; *p* < 0.05) in comparison to the older group of *Klf1* ^+/+^ mice, but no changes were observed in *Klf1* ^K74R/K74R^ mice (Figure S2M). This difference may be a sign of improvement in the age‐related cardiac systolic functions, although these RHR are still within a normal range.^[^ ^24^ ^]^ Therefore, we next examined whether KLF1(K74R) knockin impacts the adverse CV events associated with aging through a functional assessment of CV physiology with progressing age in *Klf1* ^K74R/K74R^ mice. First, the performance changes in CV function and structure, such as cardiac left ventricular (LV) mass, ejection fraction, fractional shortening, and LV myocardial thickness (LVMT) which are known indicators of CV disease in aging,^[^ ^25^ ^]^ were quantified by noninvasive echocardiography. Interestingly, as shown in **Figure** **3**A, we found that the elderly *Klf1* ^K74R/K74R^ mice maintained normal cardiac systolic function the same as young groups of animals, while the age‐related modest thickening of LVMT was also improved (Figure 3B). Conversely, the effect of aging on cardiac performance indices was observed in elderly *Klf1* ^+/+^ mice. Thus, we evaluated the extent of CV fibrosis by Masson's trichrome staining. Histologic analysis revealed that *Klf1* ^K74R/K74R^ mice exhibited less cardiac and vascular fibrosis at ages over 30 months (4.61 ± 1.48% and 25.22 ± 9.93%), and the age matched *Klf1^+/+^* mice showed a marked increase in cardiac and vascular fibrosis as anticipated (8.01 ± 4.07% and 49.89 ± 7.61%, respectively; Figure 3C,D). Based on these results, we next assessed whether KLF1(K74R) knockin was able to improve fibrotic pathogenesis in other organs. The histopathological analysis was performed on liver and kidney sections from elderly groups of animals. Consistent with the results in cardiac and liver tissues, evaluation of histopathological findings in aged animals showed that the development of hepatic fibrosis in *Klf1* ^K74R/K74R^ mice was significantly reduced to 47% compared with *Klf1* ^+/+^ mice at age over 24 months (14.84 ± 8.57% vs 31.16 ± 10.40%, *p* < 0.05) (Figure 3E). Additionally, the serum glutamic oxaloacetic transaminase (GOT) and glutamic‐pyruvic transaminase (GPT) levels of aged *Klf1* ^K74R/K74R^ mice were significantly lower (50.4 ± 6.02 U L^−1^ and 31.8 ± 5.12 U L^−1^), conversely, age‐associated elevations in GOT and GPT were determined in age‐matched *Klf1* ^+/+^ mice (78.8 ± 14.02 U L^−1^, *p* < 0.005 and 43.4 ± 5.68 U L^−1^, *p* < 0.01) (Figure 3F). Furthermore, a comparison of the morphologic features of the nephron, the functional unit of the kidney was performed. As expected, the histopathological results revealed that aged *Klf1^K74R/K74R^* mice displayed less aging‐induced kidney fibrosis than *Klf1^+/+^* mice (16.08 ± 7.30% vs 30.30 ± 10.13%, *p* < 0.05). Likewise, the extent of glomerular sclerosis, dilatation of Bowman's capsule and shrinkage of glomeruli in the renal corpuscles of *Klf1* ^K74R/K74R^ mice was also significantly lower than that in *Klf1* ^+/+^ mice (9.24 ± 7.60% vs 24.53 ± 9.77%, *p* < 0.05) (Figure 3G). These observations were further supported by urinalysis test as shown in Figure S3 (Supporting Information) and **Table** **2**. The results indicated that the levels of urine protein, urobilinogen, leukocytes, and urinary color were elevated in both *Klf1* ^K74R/K74R^ and *Klf1* ^+/+^ mice and the relative levels of these urine measurements in aged mice were comparable with young animals. We next investigated whether KLF1(K74R) knockin attenuated the incidence of spontaneous neoplasia, the commonest cause of death in laboratory mice at the end of life. To estimate the cancer incidence in vivo, we scanned animals of age 24 months for tumors by noninvasive Micro Positron Emission Tomography (MicroPET) analysis. Notably, high cancer incidence was observed in aged *Klf1* ^+/+^ mice with a spontaneous tumor incidence of 75%, while the *Klf1* ^K74R/K74R^ mice were found to be considerably resistant to tumorigenesis (about 12.5%) (Figure 3H and **Table** **3**). Together these data suggest that the progress of immune cell‐related aging, geriatric disorders, and chronic and malignant illnesses was significantly mitigated in *Klf1* ^K74R/K74R^ mice, thereby extending both healthspan and lifespan.

Figure 3Amelioration of aging‐associated tissue fibrosis and tumorigenesis in *Klf1* ^K74R/K74R^ mice. A) Echocardiographic analysis of the cardiac structure and function in young and aged *Klf1* ^K74R/K74R^ or *Klf1^+^* ^/+^ mice (*n* = 4). B) LVMT measurement among aged *Klf1* ^K74R/K74R^ and *Klf1^+^* ^/+^ mice was using standard myocardial 17‐segment mode that divided into basal, mid‐cavity, and apical region, respectively (*n* = 4). C,D) Histopathological evaluation of the extent of fibrosis in cardiac, CV and hepatic tissues (*n* = 5, 5 and 4 for each group). All tissue samples for Masson's trichrome staining were collected from animals age more than 30 months. As exemplified in photos (left panel) and statistical analysis (right panel). Whole regions of collagen‐stained tissue sections were magnified and quantitatively analyzed with the combined use of Tissue‐FAXS and Strata‐Quest software (Tissue Gnostics). Scale bar as indicated. E) Histopathological evaluation of fibrosis in liver and F) the measurement of GOT and GPT activities in blood. Samples were collected from *Klf1* ^K74R/K74R^ or *Klf1^+^* ^/+^ mice at age 24 months and the levels of GOT and GPT in serum were analyzed and compared (*n* = 5, respectively). G) Histopathologic assessment of renal tissue of aged *Klf1^K74R/K74R^* and *Klf1^+^* ^/+^ mice. Renal fibrosis was identified by carrying H&E staining in kidney samples collected from both *Klf1* ^K74R/K74R^ and *Klf1^+^* ^/+^ mice at age above 30 months (*n* = 5). Glomerular sclerosis in aging *Klf1* ^K74R/K74R^ or *Klf1^+^* ^/+^ mice was presented by the percentage (%) of Bowman's space larger than the area of the glomerulus. G: glomerulus and B: glomerular. H) Incidence of spontaneous carcinogenesis in aged mice. MicroPET scanning analysis was used to evaluate tumor incidence in *Klf1* ^K74R/K74R^ and *Klf1^+^* ^/+^ mice at age 24 month (*n* = 8), as exemplified in images (left) and statistically presented in the histogram (right). White arrowheads indicate tumor locations in *Klf1^+^* ^/+^ mice. The tumor incidence in mice was also scored by viewing with the naked eye after dissection. Significant differences were assessed by Student's *t*‐test and presented as mean ± SD and *p ≤ 0.05, **p ≤ 0.01, ***p ≤ 0.001.

**Figure:**
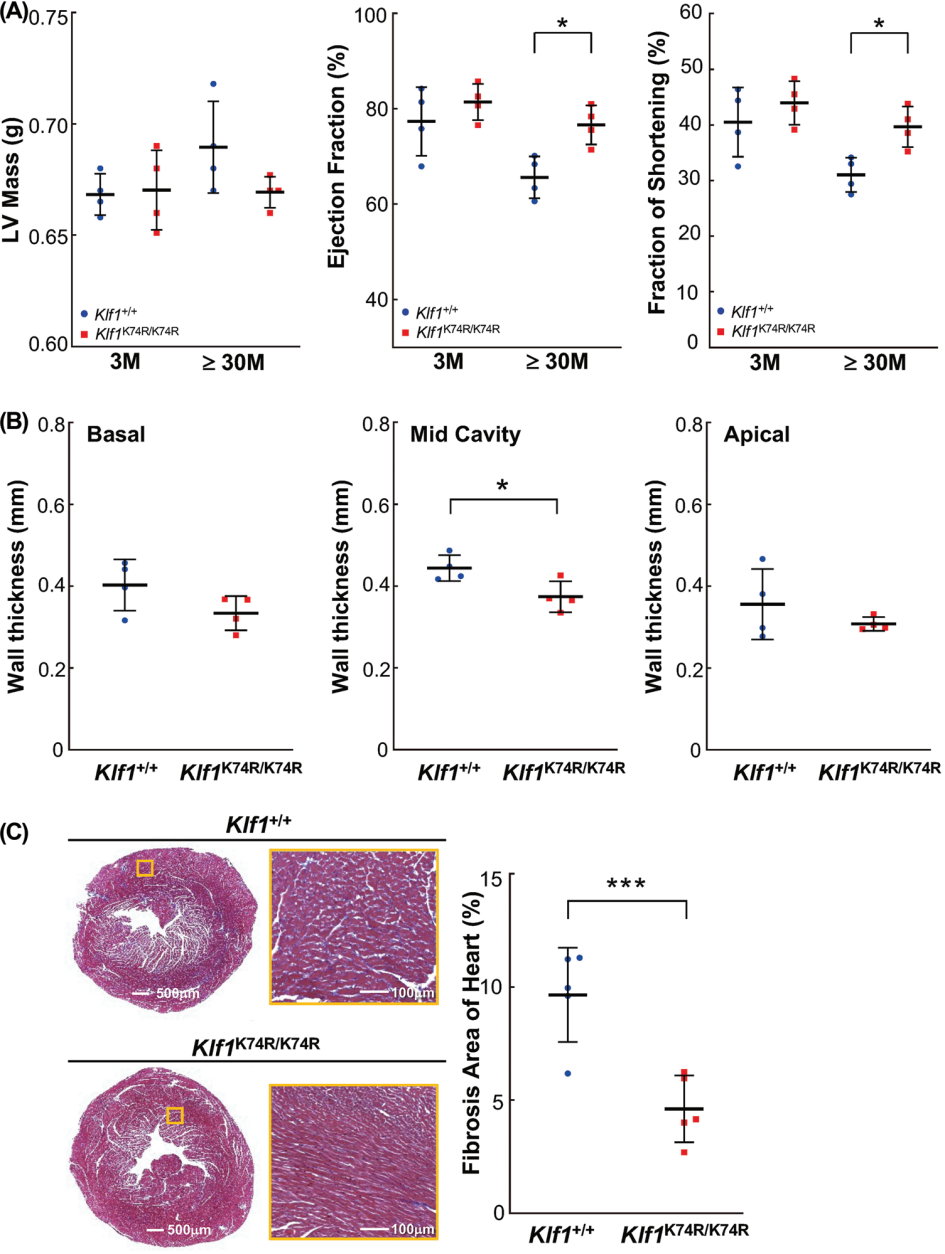

**Figure:**
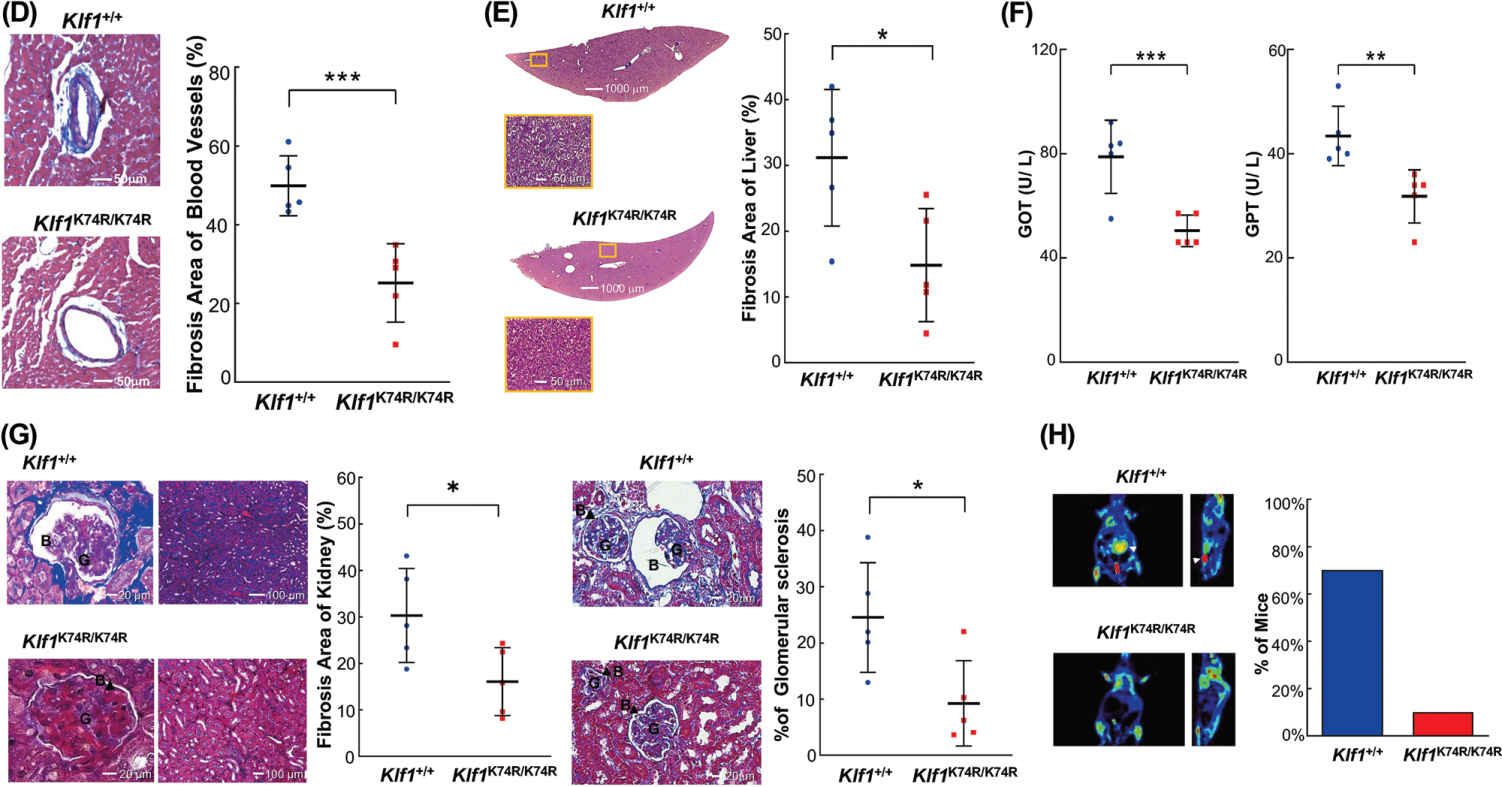

**Table 2:** Summary of urine protein, urobilinogen, urine pH, specific gravity, urine ketone, urine leukocytes, and color in *Klf1* ^+/+^ and *Klf1* ^K74R/K74R^ mice

Items	3 months		24 months	
	*Klf1* ^+/+^ (*n* = 6)	*Klf1* ^K74R/K74R^ (*n* = 6)	*Klf1* ^+/+^ (*n* = 3)	*Klf1* ^K74R/K74R^ (*n* = 3)
Protein [mg dL^−1^]	178.3 ± 826	111.7 ± 44.9	400 ± 173.2*)	100.0 ± 0.0
Urobilinogen [mg dL^−1^]	4 ± 1.1	4.8 ± 1.5	9.3 ± 2.3	6.0 ± 0.0
pH	6.3 ± 0.6	6.8 ± 0.3	6.2 ± 0.3	5.8 ± 0.3
Specific gravity	1.0 ± 0.0	1.0 ± 0.0	1.0 ± 0.0	1.0 ± 0.0
Ketone [mg dL^−1^]	7.3 ± 4.1	4.0 ± 4.7	7.3 ± 4.6	4.7 ± 4.7
Leukocyte [leu uL^−1^]	33.3 ± 20.4	33.3 ± 34.2	75.0 ± 0.0	33.3 ± 38.2
Color	Yellow	Yellow	Dark Yellow	Yellow

*) *p* ≤ 0.05

**Table 3:** Number of benign and malignant tumors as diagnosed by PET and pathological anatomy (naked‐eye)

Genotype	*Klf1* ^+/+^								*Klf1* ^K74R/K74R^							
Mice ID	1	2	3	4	5	6	7	8	1	2	3	4	5	6	7	8
*#* of Tumor (PET)	2	1	1	0	1	0	1	1	0	0	0	0	0	1	0	0
Tumor location	U	L	L		L		L	L						U		
# of Tumor (naked‐eye)	2	1	0	0	0	0	0	0	0	0	0	0	0	0	0	0
Tumor Type	H, P	B	C		C		C	C, B						H		

*Note*: U: upper abdominal cavity; Le: lower abdominal cavity; H: hepatocellular carcinoma; P: pancreatic cancer; C: colorectal cancer; B: bladder Cancer

### 2.4 KLF1(K74R) Knockin Affects Leukocyte Composition and Alleviates Age‐Associated Immune Deficiency

**Figure 4:**
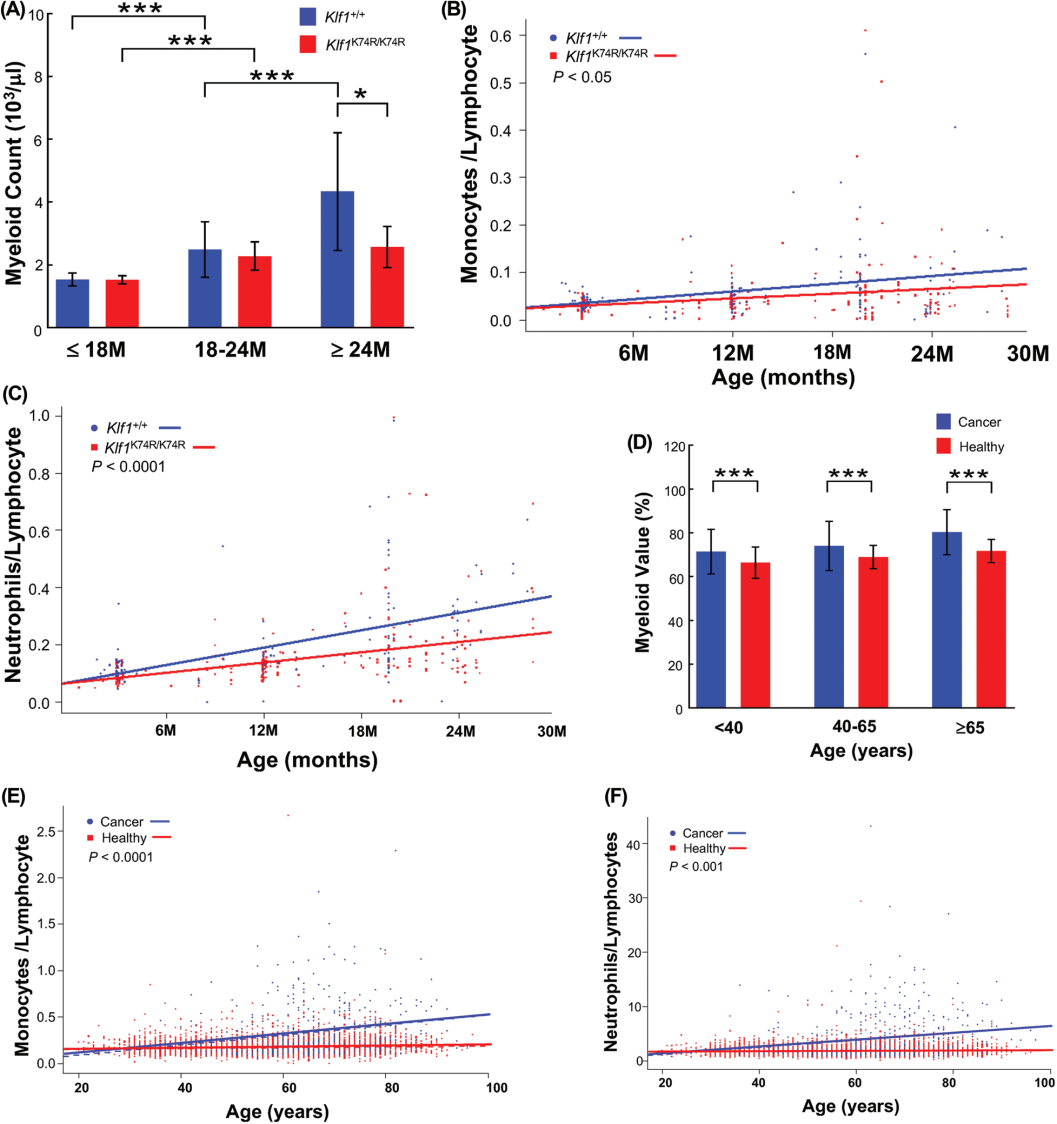
Knockin of KLF1(K74R) improves the age‐related imbalance in myeloid and lymphoid cells. The CBC data were obtained from the aged *Klf1* ^K74R/K74R^ and *Klf1^+^* ^/+^mice (*n* = 369) and volunteers (*n* = 6637), respectively. A) Comparison of frequency of myeloid cell populations between *Klf1* ^K74R/K74R^ (*n* = 150, 61, and 22) and *Klf1* ^+/+^ (*n* = 84, 42, and 10) mice at age 18, 18–24 and above 24 months, respectively. B,C) Monocyte‐to‐lymphocyte ratio (MLR) and neutrophil‐to‐lymphocyte ratio (NLR) of *Klf1* ^K74R/K74R^ and *Klf1* ^+/+^ (*n* = 233 and 136) mice, respectively. D) Comparison of frequency of myeloid cell populations between healthy volunteers and hepatocellular carcinoma (HCC) patients (*n* = 451) at age below 40, 40–65, and above 65 years. E,F) The comparison of NLR and MLR of volunteers and HCC patients, respectively. All the data were assessed by ANCOVA or Student's *t*‐test and full interaction model presented as mean ± SD and *p ≤ 0.05, **p ≤ 0.01, ***p ≤ 0.001.

Since KLF1 functions in the regulation of hematopoietic stem cell differentiation, we further analyzed the influence KLF1(K74R) knockin on myeloid/lymphoid commitment and immune compromise of *Klf1* ^K74R/K74R^ mice during the aging process. The complete blood count (CBC) test showed that there was an age‐related decrease in lymphocytes and increase in myeloid cells^[^ ^26^ ^]^ in aged *Klf1* ^+/+^ mice and this was alleviated in aged *Klf1* ^K74R/K74R^ mice (**Figure** **4**A, Figure S4 and Table S2, Supporting Information). Also, the trends in the age‐dependent elevation of neutrophil to lymphocyte ratio (NLR) and monocyte to lymphocyte ratio (MLR), the common aging‐ and disease‐associated inflammatory markers, were only mildly increased in the aged *Klf1* ^K74R/K74R^ mice group compared with *Klf1* ^+/+^ mice group (Figure 4B,C). Moreover, these results were consistent with the comparative results of CBC analysis from 6186 healthy donors and 451 cancer patients of corresponding ages that showed decrease in lymphocytes and increase in myeloid cells (Figure 4D and Figure S4I, Supporting Information). Both NLR and MLR were significantly increased in cancer patient groups compared with the healthy group (Figure 4E,F). These results imply that the influence of KLF1(K74R) knockin on the lineage commitment of hematopoietic stem cells may be associated with the age‐associated remodeling of the immune system, which attenuated the age‐associated chronic inflammation and delayed the onset of the deleterious consequences of geriatric disorders.

### 2.5 KLF1(K74R) Knockin Effectively Reduces Susceptibility to Tumorigenesis

In aged *Klf1* ^K74R/K74R^ mice, the lower cancer incidence and mild alteration in myeloid and lymphoid commitment imply that KLF1(K74R) knockin mediated lifespan extension and healthy aging may be relevant to the presence of effective cellular immunity. To explore whether the knockin of KLF1(K74R) renders *Klf1* ^K74R/K74R^ mice more able to protect against tumorigenesis, syngeneic B16F10 melanoma lung metastasis and subcutaneous tumor models were employed to evaluate the tumor susceptibility development in vivo. *Klf1* ^K74R/K74R^ mice exhibited much stronger antimetastasis activity than *Klf1* ^+/+^ mice with a dramatically reduced formation of metastatic foci in the lung (66.67 ± 41.96 vs 193 ± 54.44, *p* < 0.05) (**Figure** **5**A). The potential antitumor property of *Klf1* ^K74R/K74R^ mice was further investigated in vivo using B16 F10 melanoma and Lewis lung carcinoma (LLC) syngeneic tumor models. A comparison of tumor growth rate and tumor size after inoculation showed tumor growth was markedly delayed in *Klf1* ^K74R/K74R^ mice, while tumor weight was significantly lower than that in *Klf1* ^+/+^ mice (0.10 ± 0.04 g vs 0.67 ± 0.14 g, *p* < 0.01) (Figure 5B). Consistently, the LLC and EL4 inoculated *Klf1* ^K74R/K74R^ mice exhibited a reduction in tumor growth compared to *Klf1* ^+/+^ mice (0.17 ± 0.05 g vs 0.50 ± 0.11 g, *p* < 0.01; 460 ± 65 cm^3^ vs 156.56 ± 46.35 cm^3^, *p* < 0.05 and 0.33 ± 0.16 g vs 1.03 ± 0.66 g. Figure S5A,B, Supporting Information). Thus, to examine whether *Klf1* ^K74R/K74R^ mice have better intrinsic protection against tumorigenesis, the same metastasis assay was performed in young *Klf1* ^K74R/K74R^ mice, and the survival times after B16F10 inoculation were determined. As expected, the number of lung metastasis foci in young *Klf1* ^K74R/K74R^ mice was significantly lower than that in *Klf1* ^+/+^ mice (19.18 ± 5.64 vs 36.91 ± 9.90, *p* < 0.001), and the lower incidence of metastasis in *Klf1* ^K74R/K74R^ mice was accompanied by a small but significant increase in survival days compared to *Klf1* ^+/+^ mice (31.07 ± 4.80 vs 27.50 ± 2.77 days, *p* < 0.018) (Figure 5C). Guided by these results, we further investigated the enhancement of antitumor effects in *Klf1* ^K74R/K74R^ mice in more detail, the quantitative differences in leukocyte subsets between *Klf1* ^+/+^ and *Klf1* ^K74R/K74R^ mice was assessed utilizing high dimensional mass cytometry, CyTOF, and a multiparameter phenotyping panel that identifies 16 leucocyte markers and dissects 14 distinct leukocyte subpopulations simultaneously. As shown in Figure 5D and Figure S5C, the comparison of the viSNE map of CD45^+^ events in peripheral blood samples showed unchanged frequencies of circulating CD3^+^ T cells, CD4^+^ T cells, CD8 ^+^ T cells, and phagocyte populations between elderly *Klf1* ^K74R/K74R^ and *Klf1* ^+/+^ mice. Interestingly, we found that frequencies of effector/memory CD8^+^ T cells (1.72% ± 0.16% in *Klf1* ^+/+^ mice vs 2.72% ± 0.62% in *Klf1* ^K74R/K74R^, *p* < 0.005) and NKT cells (0.3% ± 0.07% in *Klf1^+/+^* mice vs 0.44% ± 0.11% in *Klf1* ^K74R/K74R^, *p* < 0.05) in *Klf1* ^K74R/K74R^ mice were significantly higher than those in *Klf1^+/+^* mice (**Table** **4**). Moreover, comparison of CyTOF analysis of splenocyte composition and immunohistochemical (IHC) analysis of tumor infiltrating CD8^+^ T cells and NKT cells of engrafted B16F10 melanoma tumor in lung sections from *Klf1* ^+/+^ mice and *Klf1* ^K74R/K74R^ mice revealed that the higher tumor infiltration of CD8^+^ T cells and NKT cells were significantly exhibited in *Klf1* ^K74R/K74R^ mice as compared to matched tumor tissue in *Klf1* ^+/+^ mice (Figure 5E,F and Figure S5D, Supporting Information). Furthermore, we analyzed CD3^+^ T, CD4^+^ T and CD8^+^ T cells in the peripheral lymph nodes that were mainly associated with tumor growth in the lymphatic system. The frequencies of CD3^+^ T and CD8^+^ T cells in lymph node were slightly higher but significantly in *Klf1* ^K74R/K74R^ mice (*p* < 0.05) than that in *Klf1* ^+/+^ mice (Figure S5E, Supporting Information). These results strongly suggest that the influence of KLF1(K74R) knockin, particularly in the enhancement of immune cell‐mediated immunity, plays a critical role in protection against tumorigenesis in *Klf1* ^K74R/K74R^ mice. Taken together, these results indicated that the low spontaneous tumor frequency of *Klf1* ^K74R/K74R^ mice in the period of old age was associated with strengthened intrinsic antitumor immunity via increased frequency and tumor infiltration of effector CD8^+^ T cells and NKT cells that may also contribute to prolonging lifespan in the *Klf1* ^K74R/K74R^ mice.

Figure 5Knockin of KLF1(K74R) suppresses tumor metastasis and tumorigenesis in vivo. A) The ability to resist tumor metastasis was assessed by lung colony formation assay. B16F10 melanoma cells were injected intravenously into aged *Klf1* ^K74R/K74R^ and *Klf1* ^+/+^ mice (*n* = 3). At 2 weeks postinjection, mice were sacrificed to dissect lungs for imaging and quantification of lung metastatic foci, as shown on the left and statistically presented in the histogram. B) Evaluation of tumorigenic sensitivity in aged *Klf1* ^K74R/K74R^ and *Klf1* ^+/+^ mice. B16F10 melanoma cells were subcutaneously injected into *Klf1* ^K74R/K74R^ and *Klf1* ^+/+^ mice. Tumor growth was monitored by measuring tumor volume every 2 d for a period of 2 weeks (*n* = 3, each group). Mice were sacrificed and tumors dissected for imaging and weighing. The data are represented in the form of dot histogram pairs. Results are means ± SD. Statistical significance was assessed by the two‐tailed Student's *t*‐test. C) Lung colony formation assay and survival curve of *Klf1* ^K74R/K74R^ and *Klf1* ^+/+^ mice challenged with B16F10 melanoma cells. Tumor growth kinetics were measured over 10 weeks of follow‐up. Three independent experiments were performed. Statistical significance was assessed by the log‐rank test. D) CyTOF analysis (10^7^ cells from each genotype) of 24 months old *Klf1* ^K74R/K74R^ or *Klf1* ^+/+^ mice, respectively (*n* = 5). Data were analyzed using viSNE. Applying k‐means clustering with *k*  =  10 resulted in a clear distinction between clusters in Figure S5C (Supporting Information). E,F) Tumor infiltration of CD8^+^ T and NK1.1^+^ cells in lung tumor tissues from mice injected with B16F10. The density of infiltrating CD8^+^ T cells (left panel) and NK1.1^+^ cells (right panel) was measured by IHC and analyzed using ImageJ, as determined in tumor tissue (T) and normal adjacent tissue (NAT) as control (*n* = 3, respectively).

**Figure:**
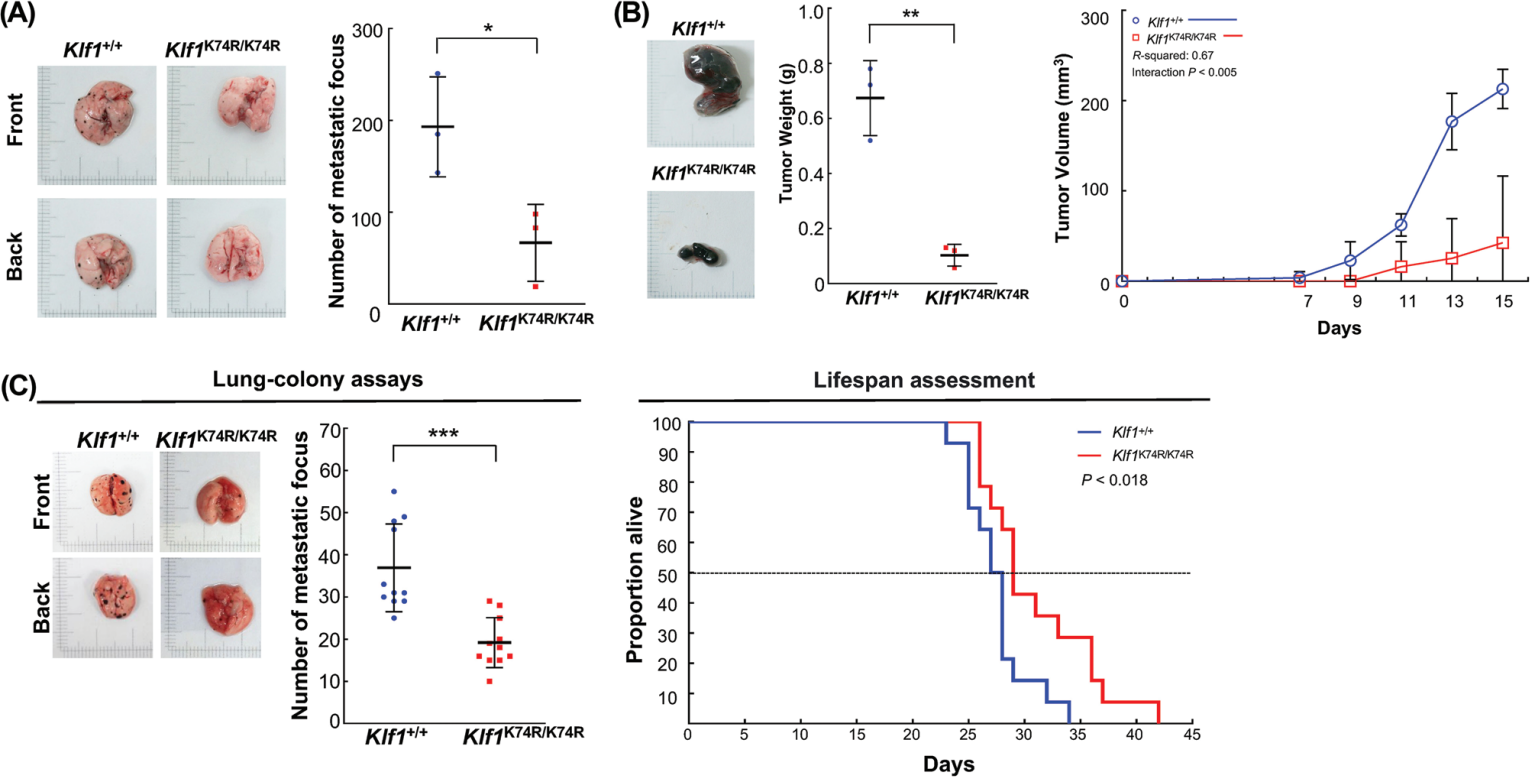

**Figure:**
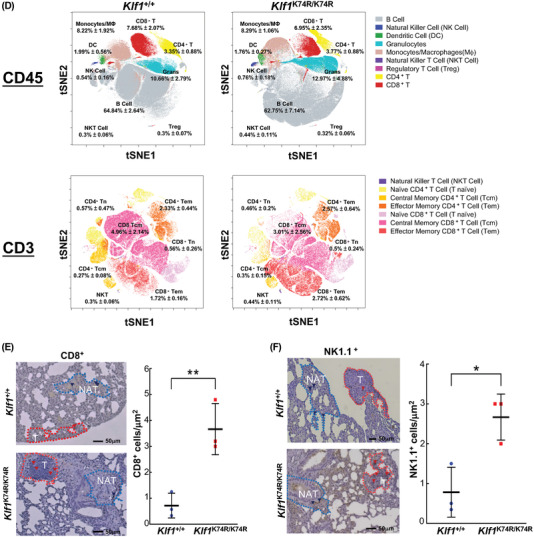

**Table 4:** The population of peripheral blood cells in 24 months *Klf1* ^+/+^ and *Klf1* ^K74R/K74R^ mice

	24 months (*n* = 5)							
Cell Types	Marker	*Klf1* ^+/+^(%)			*Klf1* ^K74R/K74R^(%)			*p* value
B cell	CD45^+^ B220^+^ CD19^+^	64.84	±	2.64	62.75	±	7.14	0.56
CD3 T Cell	CD45^+^ B220^‐^ CD19^‐^ CD3e^+^ TCRb^+^	11.48	±	3.00	11.15	±	2.84	0.86
CD4 T Cell	CD45^+^ B220^‐^ CD19^‐^ CD3e^+^ TCRb^+^ CD4^+^	3.35	±	0.88	3.77	±	0.88	0.47
Naïve CD4 T Cell	CD45^+^ B220^‐^ CD19^‐^ CD3e^+^ TCRb^+^ CD4^+^ CD44^‐^ CD62L^+^	0.57	±	0.47	0.46	±	0.20	0.64
Central Memory CD4 T Cell	CD45^+^ B220^‐^ CD19^‐^ CD3e^+^ TCRb^+^ CD4^+^ CD44^+^ CD62L^+^	0.27	±	0.08	0.30	±	0.15	0.67
Effector Memory CD4 T Cell	CD45^+^ B220^‐^ CD19^‐^ CD3e^+^ TCRb^+^ CD4^+^ CD44^+^ CD62L^‐^	2.33	±	0.44	2.57	±	0.64	0.51
CD8 T Cell	CD45^+^ B220^‐^ CD19^‐^ CD3e^+^ TCRb^+^ CD8a^+^	7.68	±	2.07	6.95	±	2.35	0.62
Naïve CD8 T Cell	CD45^+^ B220^‐^ CD19^‐^ CD3e^+^ TCRb^+^ CD8a^+^ CD44^‐^ CD62L^+^	0.56	±	0.26	0.50	±	0.24	0.75
Central Memory CD8 T Cell	CD45^+^ B220^‐^ CD19^‐^ CD3e^+^ TCRb^+^ CD8a^+^ CD44^+^ CD62L^+^	4.96	±	2.14	3.01	±	2.56	0.23
Effector Memory CD8 T Cell	CD45^+^ B220^‐^ CD19^‐^ CD3e^+^ TCRb^+^ CD8a^+^ CD44^+^ CD62L^‐^	1.72	±	0.16	2.72	±	0.62	0.01**)
Regulatory T Cell	CD45^+^ B220^‐^ CD19^‐^ CD3e^+^ TCRb^+^ CD4^+^ CD25^+^	0.30	±	0.07	0.32	±	0.06	0.64
Natural Killer T cell	CD45^+^ B220^‐^ CD19^‐^ CD3e^+^ TCRb^+^ NK1.1^+^	0.30	±	0.06	0.44	±	0.11	0.04*)
Natural Killer Cell	CD45^+^ B220^‐^ CD19^‐^ CD3e^‐^ TCRb^‐^ NK1.1^+^ CD11b^‐^	0.54	±	0.16	0.76	±	0.18	0.07
Dendritic Cell	CD45^+^ B220^‐^ CD19^‐^ CD3e^‐^ TCRb^‐^ NK1.1^‐^ CD11b^+^ CD11c^+^	1.99	±	0.56	1.76	±	0.27	0.44
Granulocytes	CD45^+^ B220^‐^ CD19^‐^ CD3e^‐^ TCRb^‐^ NK1.1^‐^ CD11b^+^ CD11c^‐^ Gr‐1^+^	10.66	±	2.79	12.97	±	4.88	0.38
Monocytes/Macrophages	CD45^+^ B220^‐^ CD19^‐^ CD3e^‐^ TCRb^‐^ NK1.1^‐^ CD11b^+^ CD11c^‐^ Gr‐1^‐^	8.22	±	1.92	8.29	±	1.06	0.94

*) *p* ≤ 0.05;

**) *p* ≤ 0.01

### 2.6 Infusion of KLF1(K74R) Knockin HSCs Potentiate Antiaging and Lifespan Extension

**Figure 6:**
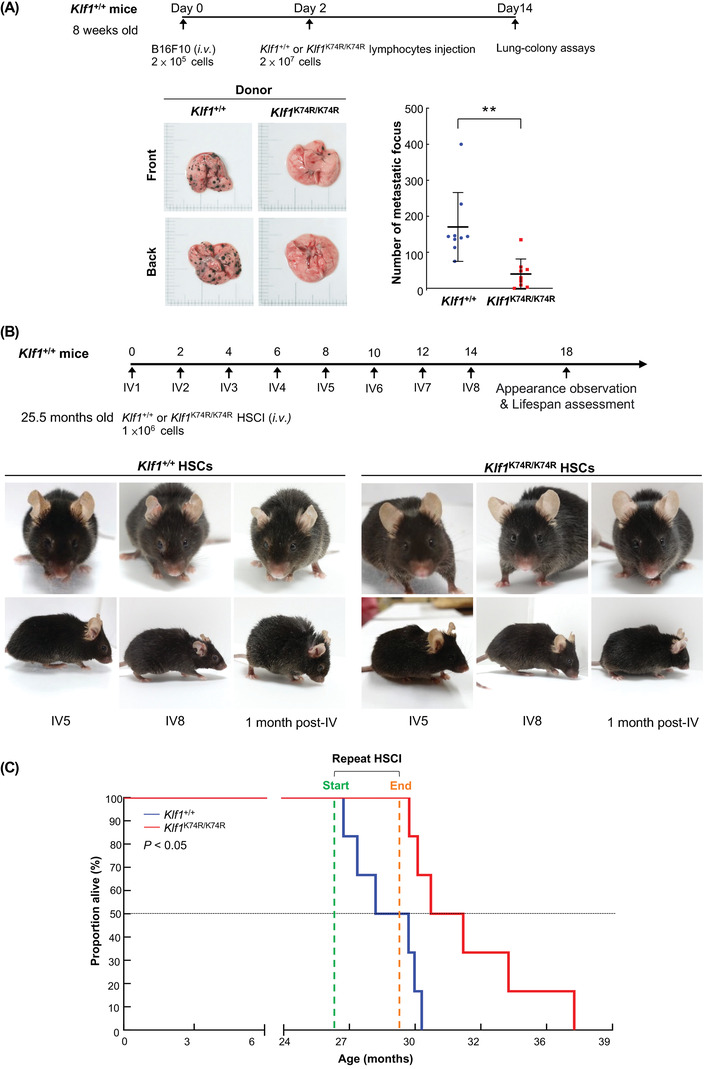
The antiaging effects of *Klf1* ^K74R/K74R^ HSC infusion. A) C57BL/6J mice were inoculated with 2 ×10^5^ B16F10 melanoma cells and then i.v. injected the leukocytes isolated from *Klf1^+^* ^/+^ or *Klf1* ^K74R/K74R^ mouse spleens at 2‐days post‐inoculation. At 2 weeks post‐injection, lungs were collected and photographed. The pulmonary melanoma foci were counted and represented as histogram (*n* = 9). Statistical significance was assessed by the two‐tailed Student's *t*‐test. B,C) Appearance observation and survival curves of aged *Klf1* ^+/+^ mice that received repeated HSC infusion. LSK‐HSCs were isolated from bone marrow of 3 months old *Klf1* ^K74R/K74R^ and *Klf1* ^+/+^ mice, respectively. 10^6^ cells were administered to aged *Klf1* ^+/+^ mice recipients (25.5 months old, *n* = 6) once every 2 weeks, a total of 8 times. Kaplan‐Meier survival curves of *Klf1* ^K74R/K74R^ LSK‐HSCs and *Klf1* ^+/+^ LSK‐HSCs was plotted from 6 recipients for each group. Statistical significance was assessed using the original method of the log‐rank test.

Our results showed that knockin of KLF1(K74R) resulted a noticeable healthspan and lifespan extension in *Klf1* ^K74R/K74R^ mice, which may be mainly related to modulation of hematopoietic and lymphatic system composition and function during the aging process. To characterize this, CD3^+^ T cells were isolated from the spleen and lymph nodes of *Klf1^K74R/K74R^* and *Klf1^+/+^* mice, respectively, and transfused into B16F10 melanoma engrafted recipient *Klf1* ^+/+^ mice (age 8 weeks) by retro‐orbital injection. As evaluated by metastatic foci formation in recipient mice, *Klf1* ^K74R/K74R^ CD3^+^ T cell transfusion exhibited fewer metastasis foci than mice that received *Klf1^+/+^* CD3^+^ T cells (**Figure** **6**A). Thus, we performed repeated infusion of *Klf1* ^K74R/K74R^ HSCs to confirm whether the antiaging effect was primarily attributed to the hematopoietic system and further evaluate the feasibility of gaining antiaging effects similar to the *Klf1* ^K74R/K74R^ mice. The Lin^−^Sca‐1^+^c‐Kit^+^ HSC (LSK‐HSC) cells were isolated from 3 months old *Klf1* ^K74R/K74R^ or *Klf1* ^+/+^ mice and administered into 25.5 months old recipient *Klf1* ^+/+^ mice, with i.v. injection every 2 weeks for up to 14 weeks. As shown in Figure 6B, *Klf1^+/+^* mice infused with *Klf1* ^K74R/K74R^ LSK‐HSCs displayed pronounced effects in amelioration of age‐related features. The visible signs of aging seen in elderly *Klf1* ^+/+^ mice were significantly improved after complete administration of *Klf1* ^K74R/K74R^ LSK‐HSCs and also in *Klf1* ^K74R/K74R^ mice, in contrast, *Klf1* ^+/+^ LSK‐HSCs infused control mice showed no improvement. Moreover, the survival curves revealed that mice that received *Klf1* ^K74R/K74R^ LSK‐HSCs had a significantly longer mean lifespan than *Klf1* ^+/+^ LSK‐HSCs infused control mice; there was about 4 months extension in lifespan (32.11 ± 3.18 months vs 28.10 ± 1.63 months, *p* < 0.05) (Figure 6C and **Table** **5**). Taken together, these results suggest that the benefit of KLF1(K74R) knockin on healthspan and longevity was hematopoietic in origin and can be simply achieved by HSCs infusion

**Table 5:** Comparative survival characteristics of *Klf1* ^+/+^ mice after HSCI

		Lifespan (months)							
Group	Genotype	Injection	Mean				Maximum	Minimum	n
Male	*Klf1* ^+/+^	*Klf1^K74R^* ^/K74R^	32.11	±	3.18	**)	37.44	29.21	6
	*Klf1* ^+/+^	*Klf1* ^+/+^	28.10	±	1.63		29.83	25.93	6

**) *p* ≤ 0.01

## 3 Discussion

Aging is commonly considered as an inevitable biological process with no effective preventative treatment. However, understanding how to reverse aging and pursue longevity has always been a preoccupation of mankind. In recent decades, several noteworthy studies concerning the plasticity of aging and the impact of potential rejuvenation factors^[^ ^27^ ^]^ have provided compelling evidence breaking the stereotype of the unpreventable process of aging and suggesting that aging may be reversible and not merely malleable. Currently, various rejuvenating and antiaging approaches in different model systems have shown that lifespan can be manipulated by interventions targeting extrinsic and intrinsic features,^[^ ^28^ ^]^ such as caloric restriction (CR),^[^ ^6b^ ^]^ blood factors,^[^ ^29^ ^]^ senescent cell elimination,^[^ ^30^ ^]^ cellular reprogramming^[^ ^31^ ^]^ and gut microbiome modulation.^[^ ^32^ ^]^

In this study, we used *Klf1* ^K74R/K74R^ mouse model to investigate the role of SUMOylated KLF1 in vivo. This K74R substitution abolished the SUMOylation of KLF1 but did not influence the activity of N‐terminal transactivation domain and C‐terminal DNA binding domain, as seen in Figure S1 (Supporting Information) and a previous study.^[^ ^20^ ^]^ Moreover, the in vitro DNA binding activity of KLF1 (Figure S1, Supporting Information) was greatly enhanced by an artificial fusion of SUMO‐1 suggesting that the phenotypic changes in *Klf1* ^K74R/K74R^ mice were completely due to the absence of SUMOylated KLF1. Intriguingly, phenotypic and blood routine examinations revealed that *Klf1* ^K74R/K74R^ mice presented an unanticipated impact upon the health status and lifespan rather than the erythropoietic system, despite its ability to act as an activator for erythropoiesis. Previous studies have suggested that SUMOylated KLF1 plays a repressive role in megakaryopoiesis, while the SUMOylation‐deficient KLF1(K74R) mutant is attenuated in its ability to repress megakaryopoiesis but without impairment of its ability to activate *β*‐globin promoter.^[^ ^20^, ^33^ ^]^ In addition, the interaction between SUMOylated KLF1 and NuRD component Mi‐2*β* implies that SUMOylated KLF1 repression of megakaryopoiesis might be regulated by the NuRD chromatin remodeling complex via silencing the expression of megakaryocytic genes.^[^ ^33^, ^34^, ^35^ ^]^ However, the possible function of the SUMOylated KLF1 in the repression of megakaryopoiesis remains to be better defined, since the steady states of the red blood cells and platelets are still maintained in *Klf1* ^K74R/K74R^ mice (Table S2, Supporting Information).

Besides the erythroid cells and megakaryocytes, KLF1 is also expressed in HSC and regulates their differentiation capability,^[^ ^34^ ^]^ in interesting correlation with the extension of the lifespan of recipient *Klf1* ^+/+^ mice by transfer of HSCs from the *Klf1^K74R/K74R^* mice (Figure 6C). In addition, KLF1 is expressed in induced pluripotent stem cell‐derived macrophages (iPSC‐DMs) as well as in erythroblastic island macrophages that assist the enucleation of terminally differentiated erythroblasts.^[^ ^35^ ^]^ Whether KLF1 plays regulatory roles in the functioning of different subsets of macrophages at the embryonic, fetal, and adult stages^[^ ^37^ ^]^ awaits future investigation. Thus, KLF1 could be expressed in specific subsets of hematopoietic cells, and the healthy longevity characteristics of the *Klf1* ^K74R/K74R^ mice most likely have resulted from the combined effects of these different types of hematopoietic/blood cells carrying the mutant KLF1(K74R) factor and consequently altered gene expression profiles as compared to the *Klf1* ^+/+^ cells. Comparative gene expression and functional analyses of the individual types of hematopoietic/blood cells from *Klf1* ^+/+^ and *Klf1* ^K74R/K74R^ mice will provide important insights into the molecular and cellular basis of the regulation of healthy longevity by KLF1.

Notably, *Klf1* ^K74R/K74R^ mouse model is distinct with respect to the numerous current aging studies including those focused on the genes involved in insulin/IGF‐1 pathway and mitochondrial functions.^[^ ^36^ ^]^ Interestingly, some aspects of KLF1(K74R)‐related antiaging effects resembled the metabolic consequences seen in dietary and pharmaceutical interventions in mice^[^ ^37^ ^]^ as shown in Figures 1, 2, 3, the lifespan, physical performance, metabolic maintenance, and progress of the age‐related disease in *Klf1* ^K74R/K74R^ mice were markedly improved under normal housing conditions that were comparable to the beneficial effects of dietary/metabolic manipulations.^[^ ^6^, ^38^ ^]^ It is noteworthy that knockin of KLF1(K74R) mutation exhibited similar antiaging effects to those seen in rapamycin treatment, intermittent fasting of periodic fasting‐mimicking diet (FMD) and inducible elimination of senescent cells,^[^ ^38^, ^39^ ^]^ such as increases median lifespan, improvement in age‐associated hematopoietic differentiation, enhancement in cognition, mitigation of age‐related inflammatory diseases, decelerate age‐related organismal deterioration and reduction of cancer incidence. In particular, the decreasing cancer incidence in *Klf1* ^K74R/K74R^ mice, also seen in the FMD treatment, is most likely due to KLF1(K74R) influencing reprogramming of the immune‐modulatory cascades of hematopoietic/blood system and improvement in CD8^+^ T‐cells and NKT cell infiltration (Figures 4 and 5 and Figure S4, Supporting Information). In comparison to FMD treatment and senescent cell ablation,^[^ ^38^, ^39^ ^]^ a noteworthy feature of the intrinsic antitumorigenesis and antitumor capability of *Klf1* ^K74R/K74R^ mice is that it can be bestowed through infusion of either lymphocytes or HSCs from *Klf1* ^K74R/K74R^ mice (Figure 6). Thus, genetically manipulating the HSCs may constitute an effective strategy for rejuvenating aging and may be more approachable and effective than current rejuvenation intervention, the CR‐related and DR‐related medication regimens, or cellular reprogramming. Finally, in line with our results, there is no gender bias in KLF1(K74R)‐related longevity, and with no obvious detrimental effects, such as restricted growth and fecundity,^[^ ^6a^ ^]^ osteoporosis, impaired wound healing, abnormal metabolism, attenuated immunity,^[^ ^40^ ^]^ tissue fibrosis^[^ ^41^ ^]^ or teratoma,^[^ ^42^ ^]^ etc., as reported in other longevity model systems. Moreover, further detailed examinations to capture subtle or unidentified adverse effects potential resulting from the KLF1(K74R) mutation are currently under investigation. On the contrary, CR treatment, DR‐related medication regimens, or cellular/genetic manipulations, although sharing common properties of longevity and greatly reducing the risk and postponing the onset of age‐related diseases are accompanied by potential adverse effects that acknowledge the limitations of their clinical application.^[^ ^3^ ^]^

In conclusion, our study suggests that *Klf1* ^K74R/K74R^ mouse model will be valuable for further investigation of molecular/cellular mechanisms underlying longevity and/or aging slowdown. It would also be useful for the identification of involved genes, factors and pathways in advance to onset of aging, while avoid the interference from aging complications. Furthermore, since KLF1 is expressed mainly in the hematopoietic/blood system, the extension of lifespan and healthspan could be achieved by repeated infusion *Klf1* ^K74R/K74R^‐HSC in mice and the orthologous *KLF1* ^K54R/K54R^‐HSC in humans. The autologous genetically manipulated mouse *Klf1* ^K74R/K74R^‐HSC or human *KLF1* ^K54R/K54R^‐HSC has the potential for development of advanced cell therapies for slow down aging and amelioration of age‐related diseases.

## 4 Experimental Section

### The Assigned Approval/Accreditation Number in Treatment of Animals

All procedures were approved by the Institutional Animal Care and Use Committees (IACUC) committee at Chang Gung Memorial Hospital Keelung branch. The IAUCU numbers are 2020092303, 2019122504, 2018122201, 2017122510, 2016092909, and 2015102301, respectively.

### Statistical and Demographic Analysis

Data are shown as means with SD unless otherwise stated. “*n*” indicates the number of animals per test group; age and sex are also noted. The Student's *t*‐test (unpaired, two‐tailed, assuming equal variance) was used for all pairwise comparisons unless specified elsewhere. All significant *p* values are < 0.05 unless otherwise stated. Demographic data were processed with Excel 2016 and GraphPad 8.0 to compute mean lifespan, median lifespan, SD, and *p* value (log‐rank test) for each cohort. The experiment was analyzed using ANOVA and the Student's *t*‐test.

## Conflict of Interest

The authors declare no conflict of interest.

## Author Contributions

P.‐C.L. and T.‐S.H. contributed equally to this work. Y.C.S., T.L.L., and C.K.J.S. designed the study and wrote the paper. Y.C.S., T.L.L., P.‐C.L., T.‐S.H., C.J.Y., M.J.L., S.M.H., X.Y.L., C.C.L., Y.H.K., C.H.L., H.L.P., J.R.C., W.J.C., N.I.Y., Y.C.C., H.C.P., S.T.J., C.C.H., G.G.L., S.S.Y., P.W.C.H., and K.J.W. executed experiments and performed the statistical analysis. All authors read and commented on the manuscript.

## Ethics Approval Statement

This study was approved by the Institutional Review Board of Chang Gung Medical Foundation and the Institutional Review Board and Human Subject Research Ethics Committee/IRB of Academia Sinica, respectively. The assigned study/project numbers are202001721A3C501, 201800289A3C606, ,201600379B0C507, AS‐IRB02‐101068, AS‐IRB01‐99054, and 99‐1401B. All experiments were carried out with the full, informed consent of the subjects.

## Clinical Trial Registration

Northeastern Taiwan Community Medicine Research Cohort (NTCMRC, ClinicalTrials.gov Identifier: NCT04839796).

## Supporting information

Supporting Information

Supplemental Movie 1

## Data Availability

Data available on request from the authors.

## References

[1] a) C. Lopez‐Otin , M. A. Blasco , L. Partridge , M. Serrano , G. Kroemer , Cell 2013, 153, 1194; 23746838 10.1016/j.cell.2013.05.039 PMC3836174

[2] B. K. Kennedy , S. L. Berger , A. Brunet , J. Campisi , A. M. Cuervo , E. S. Epel , C. Franceschi , G. J. Lithgow , R. I. Morimoto , J. E. Pessin , T. A. Rando , A. Richardson , E. E. Schadt , T. Wyss‐Coray , F. Sierra , Cell 2014, 159, 709. 25417146 10.1016/j.cell.2014.10.039 PMC4852871

[3] S. Mahmoudi , L. Xu , A. Brunet , Nat. Cell Biol. 2019, 21, 32. 30602763 10.1038/s41556-018-0206-0 PMC7653017

[4] a) J. M. Castellano , K. I. Mosher , R. J. Abbey , A. A. McBride , M. L. James , D. Berdnik , J. C. Shen , B. Zou , X. S. Xie , M. Tingle , I. V. Hinkson , M. S. Angst , T. Wyss‐Coray , Nature 2017, 544, 488; 28424512 10.1038/nature22067 PMC5586222

[5] a) L. Katsimpardi , N. K. Litterman , P. A. Schein , C. M. Miller , F. S. Loffredo , G. R. Wojtkiewicz , J. W. Chen , R. T. Lee , A. J. Wagers , L. L. Rubin , Science 2014, 344, 630; 24797482 10.1126/science.1251141 PMC4123747

[6] a) R. de Cabo , D. Carmona‐Gutierrez , M. Bernier , M. N. Hall , F. Madeo , Cell 2014, 157, 1515; 24949965 10.1016/j.cell.2014.05.031 PMC4254402

[7] J. Xu , G. Gontier , Z. Chaker , P. Lacube , J. Dupont , M. Holzenberger , Aging Cell 2014, 13, 19. 23898955 10.1111/acel.12145 PMC4326867

[8] a) J. J. Wu , J. Liu , E. B. Chen , J. J. Wang , L. Cao , N. Narayan , M. M. Fergusson , Rovira II , M. Allen , D. A. Springer , C. U. Lago , S. Zhang , W. DuBois , T. Ward , R. deCabo , O. Gavrilova , B. Mock , T. Finkel , Cell Rep. 2013, 4, 913; 23994476 10.1016/j.celrep.2013.07.030 PMC3784301

[9] D. R. Green , L. Galluzzi , G. Kroemer , Science 2011, 333, 1109. 21868666 10.1126/science.1201940 PMC3405151

[10] C. Y. Wu , Y. F. Chen , C. H. Wang , C. H. Kao , H. W. Zhuang , C. C. Chen , L. K. Chen , R. Kirby , Y. H. Wei , S. F. Tsai , T. F. Tsai , Hum. Mol. Genet. 2012, 21, 3956. 22661501 10.1093/hmg/dds210

[11] B. Conti , Cell. Mol. Life Sci. 2008, 65, 1626. 18425417 10.1007/s00018-008-7536-1 PMC2574693

[12] A. Salminen , K. Kaarniranta , A. Kauppinen , Aging 2012, 4, 166. 22411934 10.18632/aging.100444 PMC3348477

[13] J. Chang , Y. Wang , L. Shao , R. M. Laberge , M. Demaria , J. Campisi , K. Janakiraman , N. E. Sharpless , S. Ding , W. Feng , Y. Luo , X. Wang , N. Aykin‐Burns , K. Krager , U. Ponnappan , M. Hauer‐Jensen , A. Meng , D. Zhou , Nat. Med. 2016, 22, 78. 26657143 10.1038/nm.4010 PMC4762215

[14] Y. Zhu , T. Tchkonia , T. Pirtskhalava , A. C. Gower , H. Ding , N. Giorgadze , A. K. Palmer , Y. Ikeno , G. B. Hubbard , M. Lenburg , S. P. O'Hara , N. F. LaRusso , J. D. Miller , C. M. Roos , G. C. Verzosa , N. K. LeBrasseur , J. D. Wren , J. N. Farr , S. Khosla , M. B. Stout , S. J. McGowan , H. Fuhrmann‐Stroissnigg , A. U. Gurkar , J. Zhao , D. Colangelo , A. Dorronsoro , Y. Y. Ling , A. S. Barghouthy , D. C. Navarro , T. Sano , et al, Aging Cell 2015, 14, 644. 25754370 10.1111/acel.12344 PMC4531078

[15] a) O. H. Jeon , C. Kim , R. M. Laberge , M. Demaria , S. Rathod , A. P. Vasserot , J. W. Chung , D. H. Kim , Y. Poon , N. David , D. J. Baker , J. M. van Deursen , J. Campisi , J. H. Elisseeff , Nat. Med. 2017, 23, 775; 28436958 10.1038/nm.4324 PMC5785239

[16] H. Fuhrmann‐Stroissnigg , Y. Y. Ling , J. Zhao , S. J. McGowan , Y. Zhu , R. W. Brooks , D. Grassi , S. Q. Gregg , J. L. Stripay , A. Dorronsoro , L. Corbo , P. Tang , C. Bukata , N. Ring , M. Giacca , X. Li , T. Tchkonia , J. L. Kirkland , L. J. Niedernhofer , P. D. Robbins , Nat. Commun. 2017, 8, 422. 28871086 10.1038/s41467-017-00314-z PMC5583353

[17] a) I. J. Miller , J. J. Bieker , Mol. Cell. Biol. 1993, 13, 2776; 7682653 10.1128/mcb.13.5.2776-2786.1993 PMC359658

[18] A. Perkins , X. Xu , D. R. Higgs , G. P. Patrinos , L. Arnaud , J. J. Bieker , S. Philipsen , Blood 2016, 127, 1856. 26903544 10.1182/blood-2016-01-694331 PMC4832505

[19] a) Y. C. Shyu , T. L. Lee , X. Chen , P. H. Hsu , S. C. Wen , Y. W. Liaw , C. H. Lu , P. Y. Hsu , M. J. Lu , J. Hwang , M. D. Tsai , M. J. Hwang , J. R. Chen , C. K. Shen , Dev. Cell 2014, 28, 409; 24576425 10.1016/j.devcel.2014.01.007

[20] M. Siatecka , L. Xue , J. J. Bieker , Mol. Cell. Biol. 2007, 27, 8547. 17938210 10.1128/MCB.00589-07 PMC2169404

[21] C. Wang , Q. Li , D. T. Redden , R. Weindruch , D. B. Allison , Mech. Ageing Dev. 2004, 125, 629. 15491681 10.1016/j.mad.2004.07.003

[22] J. W. Hofmann , X. Zhao , M. De Cecco , A. L. Peterson , L. Pagliaroli , J. Manivannan , G. B. Hubbard , Y. Ikeno , Y. Zhang , B. Feng , X. Li , T. Serre , W. Qi , H. Van Remmen , R. A. Miller , K. G. Bath , R. de Cabo , H. Xu , N. Neretti , J. M. Sedivy , Cell 2015, 160, 477. 25619689 10.1016/j.cell.2014.12.016 PMC4624921

[23] J. Waalen , J. N. Buxbaum , J. Gerontol., Ser. A 2011, 66, 487. 10.1093/gerona/glr001 PMC3107024 21324956

[24] M. Uechi , K. Asai , M. Osaka , A. Smith , N. Sato , T. E. Wagner , Y. Ishikawa , H. Hayakawa , D. E. Vatner , R. P. Shannon , C. J. Homcy , S. F. Vatner , Circ. Res. 1998, 82, 416. 9506701 10.1161/01.res.82.4.416

[25] J. L. Fleg , J. Strait , Heart Failure Rev. 2012, 17, 545. 10.1007/s10741-011-9270-2 PMC4677819 21809160

[26] a) I. Beerman , D. Bhattacharya , S. Zandi , M. Sigvardsson , I. L. Weissman , D. Bryder , D. J. Rossi , Proc. Natl. Acad. Sci. USA 2010, 107, 5465; 20304793 10.1073/pnas.1000834107 PMC2851806

[27] M. Sinha , Y. C. Jang , J. Oh , D. Khong , E. Y. Wu , R. Manohar , C. Miller , S. G. Regalado , F. S. Loffredo , J. R. Pancoast , M. F. Hirshman , J. Lebowitz , J. L. Shadrach , M. Cerletti , M. J. Kim , T. Serwold , L. J. Goodyear , B. Rosner , R. T. Lee , A. J. Wagers , Science 2014, 344, 649. 24797481 10.1126/science.1251152 PMC4104429

[28] A. Richardson , J. Gerontol., Ser. A 2021, 76, 57. 10.1093/gerona/glaa208 32840294

[29] I. M. Conboy , M. J. Conboy , A. J. Wagers , E. R. Girma , I. L. Weissman , T. A. Rando , Nature 2005, 433, 760. 15716955 10.1038/nature03260

[30] N. S. Gasek , G. A. Kuchel , J. L. Kirkland , M. Xu , Nat. Aging. 2021, 1, 870. 34841261 10.1038/s43587-021-00121-8 PMC8612694

[31] S. Mahmoudi , A. Brunet , Curr. Opin. Cell Biol. 2012, 24, 744. 23146768 10.1016/j.ceb.2012.10.004 PMC3540161

[32] P. Smith , D. Willemsen , M. Popkes , F. Metge , E. Gandiwa , M. Reichard , D. R. Valenzano , Elife 2017, 6, e27014. 28826469 10.7554/eLife.27014 PMC5566455

[33] P. Frontelo , D. Manwani , M. Galdass , H. Karsunky , F. Lohmann , P. G. Gallagher , J. J. Bieker , Blood 2007, 110, 3871. 17715392 10.1182/blood-2007-03-082065 PMC2190608

[34] C. H. Hung , T. L. Lee , A. Y. Huang , K. C. Yang , Y. C. Shyu , S. C. Wen , M. J. Lu , S. Yuan , C. J. Shen , Int. J. Mol. Sci. 2021, 22, 8024. 34360789 10.3390/ijms22158024 PMC8347936

[35] a) S. Porcu , M. F. Manchinu , M. F. Marongiu , V. Sogos , D. Poddie , I. Asunis , L. Porcu , M. G. Marini , P. Moi , A. Cao , F. Grosveld , M. S. Ristaldi , Mol. Cell. Biol. 2011, 31, 4144; 21807894 10.1128/MCB.05532-11 PMC3187365

[36] a) M. H. Aguiar‐Oliveira , A. Bartke , Endocr. Rev. 2019, 40, 575; 30576428 10.1210/er.2018-00216 PMC6416709

[37] a) V. Azzu , T. G. Valencak , Gerontology 2017, 63, 327; 28118636 10.1159/000454924

[38] a) S. Brandhorst , I. Y. Choi , M. Wei , C. W. Cheng , S. Sedrakyan , G. Navarrete , L. Dubeau , L. P. Yap , R. Park , M. Vinciguerra , S. Di Biase , H. Mirzaei , M. G. Mirisola , P. Childress , L. Ji , S. Groshen , F. Penna , P. Odetti , L. Perin , P. S. Conti , Y. Ikeno , B. K. Kennedy , P. Cohen , T. E. Morgan , T. B. Dorff , V. D. Longo , Cell Metab. 2015, 22, 86; 26094889 10.1016/j.cmet.2015.05.012 PMC4509734

[39] a) D. J. Baker , B. G. Childs , M. Durik , M. E. Wijers , C. J. Sieben , J. Zhong , R. A. Saltness , K. B. Jeganathan , G. C. Verzosa , A. Pezeshki , K. Khazaie , J. D. Miller , J. M. van Deursen , Nature 2016, 530, 184; 26840489 10.1038/nature16932 PMC4845101

[40] D. K. Ingram , R. de Cabo , Ageing Res. Rev. 2017, 39, 15. 28610949 10.1016/j.arr.2017.05.008 PMC5565679

[41] M. J. Schafer , T. A. White , K. Iijima , A. J. Haak , G. Ligresti , E. J. Atkinson , A. L. Oberg , J. Birch , H. Salmonowicz , Y. Zhu , D. L. Mazula , R. W. Brooks , H. Fuhrmann‐Stroissnigg , T. Pirtskhalava , Y. S. Prakash , T. Tchkonia , P. D. Robbins , M. C. Aubry , J. F. Passos , J. L. Kirkland , D. J. Tschumperlin , H. Kita , N. K. LeBrasseur , Nat. Commun. 2017, 8, 14532. 28230051 10.1038/ncomms14532 PMC5331226

[42] a) M. Abad , L. Mosteiro , C. Pantoja , M. Canamero , T. Rayon , I. Ors , O. Grana , D. Megias , O. Dominguez , D. Martinez , M. Manzanares , S. Ortega , M. Serrano , Nature 2013, 502, 340; 24025773 10.1038/nature12586

