# Supplementary material for: Genetic Disruption of KLF1 K74 SUMOylation in Hematopoietic System Promotes Healthy Longevity in Mice
Source: Adv Sci (Weinh). 2022 Jul 13;9(25):2201409. doi: 10.1002/advs.202201409 (PMC9443461; doi:10.1002/advs.202201409)
Supplement: Supplementary file 1 — Supporting Information [file ADVS-9-2201409-s001.pdf]

ADVANCED SCIENCE
Open Access

Supporting Information

for *Adv. Sci.*, DOI 10.1002/advs.202201409

Genetic Disruption of KLF1 K74 SUMOylation in Hematopoietic System Promotes Healthy Longevity in Mice

Yu-Chiau Shyu, Po-Cheng Liao, Ting-Shou Huang, Chun-Ju Yang, Mu-Jie Lu, Shih-Ming Huang, Xin-Yu Lin, Cai-Cin Liou, Yu-Hsiang Kao, Chi-Huan Lu, Hui-Ling Peng, Jim-Ray Chen, Wen-Jin Cherng, Ning-I Yang, Yung-Chang Chen, Heng-Chih Pan, Si-Tse Jiang, Chih-Chin Hsu, Gigin Lin, Shin-Sheng Yuan, Paul Wei-Che Hsu, Kou-Juey Wu, Tung-Liang Lee* and Che-Kun James Shen**

Supporting Information

Genetic Disruption of KLF1 K74 SUMOylation in Hematopoietic System Promotes Healthy Longevity in Mice

Yu-Chiau Shyu [*], Po-Cheng Liao [#], Ting-Shou Huang [#], Chun-Ju Yang, Mu-Jie Lu, Shih-Ming Huang, Xin-Yu Lin, Cai-Cin Liou, Yu-Hsiang Kao, Chi-Huan Lu, Hui-Ling Peng, Jim-Ray Chen, Wen-Jin Cherng, Ning-I Yang, Yung-Chang Chen, Heng-Chih Pan, Si-Tse Jiang, Chih-Chin Hsu, Gigin Lin, Shin-Sheng Yuan, Paul Wei-Che Hsu, Kou-Juey Wu, Tung-Liang Lee [*] and Che-Kun James Shen [*]

Abbreviation
Supplementary Result
Experimental Section/Methods
Supplementary figure legends
Supplementary figures
Supplementary Table
Cover figure
Movie files

Abbreviation

Klf1: Krüppel-Like Factor 1; *Eklf:* Erythroid Krüppel-Like Factor ;*Gcdh*: glutaryl-Coenzyme A dehydrogenase; *Dnase2a*: deoxyribonuclease II alpha; *Mast1*: microtubule associated serine/threonine kinase 1; *Syce2*: synaptonemal complex central element protein 2; TCHO: Total cholesterol; TG: Triglyceride; HDL: High-density lipoprotein; GOT: Glutamic Oxaloacetic Transaminase; GPT: Glutamic Pyruvic Transaminase; TBIL: Total bilirubin; IGF-1: Insulin-like growth factor 1; TOR: Target of rapamycin; VO_2: Oxygen consumption; VCO_2: Carbon dioxide production; RER: Respiratory exchange ratio; ECG: Echocardiography; LV mass: Left ventricular mass; LVEF: Left ventricular ejection fraction; PET: positron-emission tomography. MRI: Cardiac magnetic resonance imaging; NLR: neutrophil to lymphocyte ratio; MLR: monocyte to lymphocyte ratio; LVIDd: left ventricular internal diameter in diastole; LVIDs: left ventricular internal diameter in systole; CyTOF: Cytometry by time of flight.

Supplementary Result

The K54R mutation of KLF1 is very rare in humans. We have inspected two human genome databases, gnomAD (n = 123136) and ExAC (n = 94998) and sequenceing 193 participant specimens which recruited from Department of General Surgery, Chang Gung Memorial Hospital, Keelung branch, Department of Hematology Oncology, Chang Gung Memorial Hospital, Keelung branch, Taiwan Blood Services Foundation, and Northeastern Taiwan Community Medicine Research Cohort (NTCMRC, ClinicalTrials.gov Identifier: NCT04839796) and sample preservation was conducted in the Community Medicine Research Center of Chang Gung Memorial Hospital, Keelung branch. Thus far, only one subject has been recorded with the mutation (in ExAC) which was also registered in the dbSNP with ID rs759212421.

Experimental Section/Methods

Treatment of Animals

C57BL/6J (B6), B6(Cg)-Tyr^{c-2J}/J (B6-albino mice) were obtained from the Jackson Laboratory (Bar Harbor, ME, USA). Mice were bred and housed in specific pathogen-free (SPF) and Association for Assessment and Accreditation of Laboratory Animal Care (AAALAC) certified barrier facilities. All procedures were approved by the Institutional Animal Care and Use Committees (IACUC) committee at Chang Gung Memorial Hospital and Academia Sinica. Animals included in longevity studies were allowed to die naturally, and mice included in tumor metastasis survival studies were allowed to die of disease progression.

Electrophoretic mobility shift assay (EMSA)

The electrophoretic mobility shift assay (EMSA) was performed with 5 μg of nuclear extracts from transfected 293T cells (ATCC, Manassas, VA,USA) with binding buffer containing 20 mM HEPES (pH 7.5, Sigma Aldrich, St. Louis, MO, USA), 40 mM KCl (Sigma Aldrich), 2.5 mM $MgCl_2$ (Sigma Aldrich), 0.01% NP40 (ThermoFisher Scientific, Waltham, MA, USA), 5 mM EDTA (pH 8.0, J.T. Baker, Phillipsburg, NJ, USA), 1 mM $MgCl_2$, 5 mM DTT (Sigma Aldrich), 0.1 μg BSA (ThermoFisher Scientific), 0.1 mM PMSF (Sigma Aldrich), 1 mM DTT(Sigma Aldrich), 5% glycerol (J.T. Baker) and 0.2 μg of poly dI-dC (deoxyinosine-deoxycytidylic, Sigma Aldrich) and all procedures were followed as described previously.[1] The ^{32}P-labeled DNA probe was prepared by annealing mouse β-major globin CACCC box containing oligonucleotides, 5'-TAGAGCCACACCCTGGTAAG-3' and 5'-CTTACCAGGGTGTGGCTCTA-3' and labeled using Klenow enzyme with [α-32P] dATP. The KLF1 binding was confirmed by super-shift assay with Monoclonal ANTI-FLAG M2 antibody (Cat. No. F3165; Sigma Aldrich).

Generation of KLF1(K74R) Knockin Mouse Model

KLF1(K74R) knockin mice were generated in collaboration with Dr. Si-Tse Jiang of the Transgenic Core Facility at the Institute of Molecular Biology, Academia Sinica. Bacterial artificial chromosome (BAC) clone RP24-319P23 (Children's Hospital Oakland Research Institute, CA) was used in a multi-step polymerase chain reaction (PCR) to delete 50 bp in the first intron of the mouse *Klf1/Eklf* gene, Chr8: 84,901,928-84,905,291 (GRCm38:CM001001.2). The map of the mouse *Klf1* gene locus of the loxP-PGK-gb2-neo-loxP K74R retroviral vector was used to knockin the K74R mutation into exon 2 (E2) of the mouse *Klf1* gene. The protein-encoding portion of E2 was replaced (asterisks) by loxP-PGK-gb2-neo-loxP K74R retroviral

vector and a neomycin cassette (neo, open box) flanked by lox P sites (black box). The region of interest containing the K74R mutation was inserted into the PGK-gb2-neo-loxP gene targeting cassette **(Figure 1A)** via homologous recombination. The modified PGK-gb2-neo-loxP construct contained a negative selection marker for embryonic stem cell (ESC) targeting. The targeting construct was delivered into B6 ECSs via electrophoresis. The copy number of the native *Klf1* gene was screened in 375 ES cell colonies via dPCR (digital PCR instrument) standardized to two nearby reference genes, namely *Nfix* and *Fbwx9* **(Figure S1B)**. The integrity of the three gene loci used in dPCR was preliminarily verified via PCR. The loss of a copy of the native *Klf1* gene indicated successful gene targeting **(Figure S1C)**. Thereafter, chimeras were generated with the positive clones and backcrossed with B6(Cg)-Tyr^{c-2J}/J, or B6-albino mice to produce *Klf1*$^{PGK/+}$ mice. Germline transmitting F1 *Klf1*$^{PGK/+}$ mice were crossed with EIIa-Cre expressing mice to excise the neomycin selection marker through lox-P recombination, generating heterozygous *Klf1*$^{K74R/+}$ mice and another B6 line of mice, line #86 *Klf1*$^{K74R/K74R}$ **(Figure S1D)**. The *Klf1*$^{K74R/+}$ heterozygotes were then intercrossed to generate homozygous *Klf1*$^{K74R/K74R}$ knockin mice and *Klf1*$^{+/+}$ mice **(Figure 1)**.

Primers used in the establishment of *Klf1*$^{K74R/K74R}$ mice are listed below:

**Primer name**	**Sequence**
Klf1 5FA	5'- AGGCAGAAGAGAGAGAGGAGGC -3'
Klf1 P5RA	5'- GCGGCGCGATAACTTCGTAT -3'
Klf1 5RA	5'- TTAGAATTCTGCTTCCTGTTGGA -3'
Gt *Klf1* d 5'	5'- CCTATTTCTCCAACAGGAAGCA -3'

Gt *Klf1* PGK5’	5’- GTTATGCGGCCCTAGTGATTTA -3’
Gt *Klf1* 3’	5’- CTGGCCCTCAAACAACCCTG -3’
dPCR *Klf1* F	5’- CAAGGTGAGGCCTATTTCTCCAA -3’
dPCR *Klf1* R	5’- GACCTTTGTCTCTATGAGCCTAGTC -3’
dPCR *Klf1* Probe	5’- CAGGAAGCAGAATTC -3’
dPCR *Nfix* F	5’- ATGGCCCAATGACGTGGAT -3’
dPCR *Nfix* R	5’- GAGGCCACCGGAAGCA -3’
dPCR *Nfix* Probe	5’- CCATGCCCATACCTGC -3’
dPCR *Fbxw9* F	5’- CGAGCCAATAGCGTCCTACAG -3’
dPCR *Fbxw9* R	5’- TGCACTCCTTCTCTGAGACACA -3’
dPCR *Fbxw9* Probe	5’- AAGGGTCACCCGCCTGC -3’
PCR *Klf1* F	5’- AGGCAGAAGAGAGAGAGGAGGC -3’
PCR *Klf1* R	5’- TTAGAATTCTGCTTCCTGTTGGA -3’
PCR *Nfix* F	5’- TGGAGCCTGGGTGAACAAGG -3’
PCR *Nfix* R	5’- AGACCATGATGGGGCGCCTA -3’
PCR *Fbxw9* F	5’- AGCCATCACCCCAGCCTTGT -3’
PCR *Fbxw9* R	5’- GGAAGGGCAACCAGCGTTCT -3’

Mouse Genotyping

Tail and toe clippings (post-natal day 14) were digested overnight in direct lysis buffer [0.05 M Tris (VWR Life Science, Radnor, PA, USA), 0.1M EDTA (J.T. Baker), and 0.5% SDS (Panreac, Barcelona, Spain)] containing proteinase K at 55°C. Protein was precipitated and removed with 7.5 M Ammonium acetate (VWR International Ltd, Lutterworth, UK). Briefly, 0.75 mL of isopropanol (J.T. Baker) was added to precipitate the DNA, and the precipitate was washed with 75% ethanol (J.T.

Baker). DNA pellets were air-dried for 10-20 minutes at 25°C and resuspended in ddH$_2$O. Extracted DNA was then used for PCR genotyping via the Invitrogen Taq DNA Polymerase kit (ThermoFisher Scientific) according to the manufacturer's specifications. All primers were obtained from Qiagen (Hilden, Germany).

The PCR program consisted of 4 min at 94°C, 28 cycles of 94°C for 30 sec, 55°C for 40 sec, 72°C for 40 sec, with a final extension of 72°C for 7 min. Expected band sizes were 312 bp and 278 bp for the *Klf1*$^{K74R/K74R}$ and *Klf1*$^{+/+}$ genotype, respectively **(Figure S1C)**. The sequences of representative PCR products are shown in Figure S1D. The Cre genotype was also determined similarly. Primers used for genotyping were: *Klf1* (Kin5': sense 5'-TGGGGCCAGTGTGAGTGTGT-3' and Kin3': antisense 5'-GCTCAAAGCCTGGAAATCCCA-3') and *Cre* (sense 5'-CCACGACCAAGT GACAGCAATG-3' and antisense 5'-CAGAGACGGAAATCCATCGCTC-3').

RNA Extraction

Total RNA was extracted via the Invitrogen™ Trizol reagent (ThermoFisher Scientific) and reverse-transcribed with the SuperScript™ III First-Strand Synthesis System (ThermoFisher Scientific) according to the manufacturer's protocol.

Semiquantitative RT-PCR

Mouse *Klf1*, *εy globin*, *βmajor globin*, and *β-Actin* mRNA levels were determined by semiquantitative RT-PCR. The primers used for each gene were: *Klf1* (sense 5'-GCGCCACAGTACCAAGGCCACT-3' and antisense 5'-GAGCGAGCGAACCTCCAGTCAC-3'), *εy globin* (sense 5'-AATGGCCTGTGGAGTAAGGTC-3' and antisense 5'-ACTTGTGGGACAGCGCAGTG-3'), *βmajor globin* (sense

5’-GCACCTGACTGATGCTGAGA-3’ and antisense 5’-CCAAGTGATTCAGGCCATC-3’), and *β-Actin* (sense 5’-CCTCCCTGGAGAAGAGCTATGA-3’ and antisense 5’-CCACCGAT CCACACAGAGTACTT-3’).

The semiquantitative RT-PCR was conducted with the following program: 1 cycle at 94°C for 5 min, followed by n cycles at 94°C for 30 sec, Annealing at T_a for 40 sec, and 72°C for 30 sec, where n is defined separately for each gene. Cycle numbers and T_a used for each gene are listed below:

**Gene name**	**Cycle numbers**	**T_a**
Klf1	25, 27, 29	60°C
εy globin	19, 21, 23	55°C
βmajor globin	16, 18, 20	52°C
β-Actin	19, 21, 23	55°C

RT-qPCR

The qPCR assays were performed using the Applied Biosystems 7500 Real-Time PCR System (ThermoFisher Scientific) with Applied Biosystems TaqMan gene expression assays Time PCR System (Thermo Fisher Scientific), and the expression of *Klf1* (ID: Mm04208330_g1 and Mm00516096_m1) was normalized to *Gapdh* (ID: Mm99999915_g1) or *β-Actin* (ID: Mm00607939_s1) expression in the same samples. Data are presented as histograms, and each bar represents the mean ± SD of three technical repeats.

Gene expression Profiling by Affymetrix RNA Microarray Array Hybridization

The total bone marrow RNAs from *Klf1*$^{K74R/K74R}$ and *Klf1*$^{+/+}$ mice were then isolated with Trizol reagent (Invitrogen, Carlsbad, CA, USA) and were subjected to genome-scale gene expression profiling using the Mouse Genome Array 430A 2.0 (Affymetrix, Inc., Santa Clara, CA, USA). The standard MAS5.0 method was applied to normalize the gene expression data. The gene expression values were log-transformed for later comparative analysis. Statistical analysis was carried out using R 3.0.2 language (R Development Core Team, 2013, http://www.R-project.org, accessed on 21 July 2021) , Hierarchical Clustering and heatmap analysis was conducted with the open source software Multi-experiment Viewer (MeV 4.9.0, https://sourceforge.net/projects/mev-tm4/files/mev-tm4/).

Western blot

Polyclonal rabbit KLF1 antibodies were generated in-house as detailed previously.[2] The antibody was purified from rabbit sera using an affinity column, then concentrated, and stored at -20°C.[2] Mouse anti-actin monoclonal antibodies were purchased from Sigma Aldrich (Cat. No. A5441). Western blotting procedures were described in our previous publications.[3]

Kaplan-Meier Survival Curves

The curves were generated by GraphPad 8.0 (Prism, San Diego, CA, USA). Demographic data were processed with Excel 2016 and GraphPad 8.0 to compute mean, median, maximum, minimum, youngest 10% and oldest 10% lifespans, as well as SD, percent alive, and P values for each group. Maximum and minimum lifespans were defined as the age of death of oldest and youngest mice in each group.

Body Weight and Body Temperature

Body weight and body temperature were measured during experiments and at sacrifice using an electronic scale and a rectal probe thermometer (Model No: TH-5) (Physitemp, New Jersey, USA). The detailed measurement procedure is described below. The mice were taken out of the cage and placed on an electronic scale to record the body weight value. The core body temperature of mice was detected by inserting the rectal probe into the rectum smoothly. The rectal probe was sterilized with 75% ethanol before each measurement. The inserted depth of probe was about 2 - 3 cm for 3 seconds to measure the temperature.

Blood Pressure

The blood pressure and pulse of mice were measured using an arterial pressure analysis system (BP-2000 Series II, Kent Scientific, Torrington, CT), and a non-invasive tail cuff system, in 3, 24, and over than 30 months old mice. The tail-cuff was first inspected for leakage and the pressure was calibrated, adjusting the temperature of the platform to 38°C. Mice were loaded onto the platform and covered with a magnetic restrainer, then the tail was passed through the cuff and pulse sensor before carefully fixing the distal part of the tail onto the platform. At least three complete measurements of basal systolic blood pressure (SBP), diastolic blood pressure (DBP), and heart rate (0 hour) of mice were recorded.

Body Composition

The body composition of the animals was determined via nuclear magnetic resonance (NMR) (Minispec Live Mice Analyzer LF50 TD-NMR, Bruker Corp., Billerica, MA, USA) by the Taiwan Mouse Clinic and Taiwan Animal Consortium. The components

including body weight, body fat, body lean, free fluid, and total water were performed and read three times.

Indirect Calorimetry, Food Consumption, and Water Consumption

Indirect calorimetry was performed by the Taiwan Mouse Clinic and Taiwan Animal Consortium to assess metabolic rates of test animals. O_2 consumption (VO_2) and CO_2 production (VCO_2) were determined via the LabMaster Calorimetry Module calorimetry system (TSE-systems GmbH, Berlin, Germany). Animals were allowed to acclimate to the calorimetry chambers for 24 h, and after the acclimation period, the VO_2 and VCO_2 were measured every 30 min for 48 h, but only data from the first 24 h were used for analysis. Respiratory exchange ratio (RER) and heat output (H) were extrapolated via methods described by Weir.[4] Animals were fed *ad libitum* during the procedure, and daily food and water intake values were recorded for a sub-group of animals throughout the assay.

Grip Strength Tests

Grip strength of *Klf1*$^{K74R/K74R}$ and *Klf1*$^{+/+}$ mice was measured with the MK-380CM/R (Muromachi, Japan) grip strength meter at the Taiwan Mouse Clinic and Taiwan Animal Consortium. Mice were allowed to grab onto the apparatus with their front paws and were pulled horizontally, via their tails, by the operator with consistently increasing force. The force at which grip failure occurred was recorded as the respective grip strength of each subject. Each animal was subjected to 5 trials with 30 sec of rest between each test and each data point was normalized to the body weight of the subject.

Rotarod and Water Maze Tests

Rotarod experiments were conducted by the Taiwan Mouse Clinic and Taiwan Animal Consortium. The rotarod test was performed on a Rota-Rod 47600 automatic rotarod (Ugo Basile Biological Research Apparatus Company, Comerio, Italy). In the training session, the mouse was placed on the rod at 0 rpm for 60 sec and three additional sessions at 4 rpm for 60 sec to allow the animal to acclimate to the procedure. The mice were then placed on the rod at a speed that accelerated from 4 to 40 rpm over 300 sec. The time and rpm at which the mice fell off the rotarod were recorded.

Water maze (Morris Water Maze; MWM; EthoVision XT, Wageningen, the Netherlands) experiments were conducted in a circular arena, 150 cm in diameter. The arena was surrounded by 60 cm tall walls and filled to 30 cm with water. A visual reference was visible to the mice above the perimeter walls. Mice were inserted at the perimeter of the arena directly under the visual cue and 90, 180, and 270 degrees from the visual cue. An invisible platform (28 cm tall) was placed in a set spot in the arena, and the time each mouse took to reach the platform was recorded as the escape latency. After the hidden platform tasks, a visible platform (32 cm tall) was placed in the arena, and the study was repeated to exclude differences in vision, swim speed, and movement among the different mice. Animals were subjected to four trials per session and two sessions a day. A complete test consisted of five sessions.

Echocardiogram

Echocardiograms were conducted in the Keelung Chang Gung Memorial Hospital radiology department using an Automatic Echocardiogram. Mice were sedated with

isoflurane for the procedure. Left Ventricular Mass (LVM), Ejection Fraction (EF), and Fraction of Shortening (FS) were calculated with the following parameters:

LVM = 1.05 [(IVS + LVID + LVPW)^3 – (LVID)^3]

EF% = 100 × [(LVIDd3 – LVIDs)^3/(LVIDd)^3]

FS% = 100 × [(LVIDd – LVIDs)/(LVIDd)]

*IVS: interventricular septal thickness, LVID: left ventricular internal diameter, LVPW: left ventricular posterior wall thickness, LVIDd, left ventricular internal diameter in 12iástole, LVIDs, left ventricular internal diameter in systole.

Histopathology

Mice were anesthetized by isoflurane, and their hearts were perfused with saline before surgical removal. Mouse hearts, livers, and kidneys were removed and fixed in paraffin, and tissue was sectioned into 3 mm slices and drop fixed in a 10% formalin solution for 48 h at room temperature. The slices were then deparaffined with xylene and rehydrated through an ethanol gradient. Rehydrated samples were stained with the hematoxylin and eosin (H&E) and Trichrome Staining (Masson) Kit (Sigma Aldrich) according to the manufacturer's instructions. Stained slides were imaged in a TissueFAXS system (TissueGnostics, Vienna Austria, Austria) and analyzed using the StrataQuest Analysis Software (TissueGnostics).

PET and Necropsy of 24 Months Old Mice

PET scans were performed MicroPET R4 (Concorde Microsystems, Cat. No. Ge-68, Sanders Medical, TN, USA) system in Taipei Veterans General Hospital. Full specification and system performance can be found in Knoess et al..[5] Mice were anesthetized with isoflurane and injected with 18F-FDG (100 Ci) 30 min before

imaging. Subsequently, 18F-FDG PET was acquired in the 2D mode for 10 min, and scans were reconstructed via ordered subsets expectation maximization (OSEM) (128 × 128-pixel image matrix, 16 subsets, 4 iterations). The resultant image had a voxel size of 0.42 mm × 0.42 mm × 1.21 mm and the field of view of 108.2 mm × 108.2 mm × 76.32 mm. PET scans were analyzed by radiologists at the Keelung Chang Gung Memorial Hospital; as per standard practice, the identities of the animals were blinded to the examining doctor. The number of tumors identified via the PET scan was recorded. The mice were then sacrificed via cervical dislocation, the peritoneal cavity of the animal was opened, and the number of visible tumors was noted.

Comprehensive Biochemical Serum Analysis

A comprehensive blood analysis was performed by the Taiwan Mouse Clinic and Taiwan Animal Consortium. Serum was obtained from test animals via submandibular blood collection. Total cholesterol (TCHO), triglyceride (TG), high-density lipoprotein (HDL), aspartate transaminase (GOT), alanine transaminase (GPT), blood urea nitrogen (BUN), and blood creatinine (CRE) levels were measured using an automatic clinical chemistry analyzer (Fuji Dri-Chem 4000i, Fujifilm, Tokyo, Japan) using the corresponding test strip.

Insulin, IGF-1, and Glucose Tolerance Test

Serum insulin and IGF-1 were determined by ELISA kits (EMINS, Thermo Fisher Scientific) according to the manufacturer's instructions. For the oral glucose tolerance test, mice were fasted for 12 h (22:00 to 10:00 the following day) before glucose dosing. Blood was drawn from the tail vein and measured with the Onetouch Ultra test strip (Lifescan, Milpitas, CA, USA) immediately before dosing. Mice were then

administered with 1.5 mg/g body weight of glucose via gavage feeding of 0.3 mg/mL glucose solution. Blood glucose measurements were taken at the 15, 30, 60, 90, and 120 min after glucose administration using the Onetouch Ultra test strip (Lifescan).

Urine Analysis

Urine was collected from mice and the analysis was performed by the Taiwan Mouse Clinic and Taiwan Animal Consortium. The parameters such as urinary protein, urobilinogen, pH, specific gravity, ketone, and leukocytes of using the Aution *Eleven* versatile urine analysis system AE-4020 (Arkray, Minneapolis, MN, USA) by dual-wavelength reflectance method and the wavelengths (LED: 430, 565, 635 and 760 nm).

Participant Recruitment for CBC

Between October 2014 and September 2019, 6637 participants were recruited from the Northeastern Taiwan Community Medicine Research Cohort (NTCMRC, ClinicalTrials.gov Identifier: NCT04839796) and sample preservation was conducted in the Community Medicine Research Center of Chang Gung Memorial Hospital, Keelung branch. The Institutional Review Board (IRB) number are 202001721A3C501, 201800289A3C606 and 201600379B0C507, respectively.

Complete Blood Count (CBC)

The blood was collected during experimental (cheek blood sampling) and at sacrifice (cardiac puncture) in a blood collection tube with EDTA. Each 200 μL whole blood sample was analyzed on a ProCyte Dx Hematology Analyzer (IDEXX, Westbrook, ME, USA). The measurements of neutrophils, lymphocytes, monocytes, eosinophils,

basophils, white blood cell count, and percentages of each cell type were performed by the Taiwan Mouse Clinic and Taiwan Animal Consortium. The participant recruitment and sample preservation were carried out from the Northeastern Taiwan Community Medicine Research Cohort (NTCMRC, ClinicalTrials.gov Identifier: NCT04839796). This study has been approved by the Institutional Review Board of Chang Gung Memorial Hospital.

Human KLF1 sequencing

Between January 2011 and April 2022, 193 participants were recruited from the Department of General Surgery, Chang Gung Memorial Hospital, Keelung branch., Department of Hematology Oncology, Chang Gung Memorial Hospital, Keelung branch., Taiwan Blood Services Foundation, and Northeastern Taiwan Community Medicine Research Cohort (NTCMRC, ClinicalTrials.gov Identifier: NCT04839796) and sample preservation was conducted in the Community Medicine Research Center of Chang Gung Memorial Hospital, Keelung branch. The blood sample was collected in a blood collection tube with EDTA and total RNAs was isolated by Trizol reagent (Invitrogen, Carlsbad, CA, USA) through RNA extraction procedure. The primers used for amplicon of human *KLF1* gene are sense 5’-GTGTTGGGGAAGTGGGACAGAC-3’ and antisense 5’-GGAAGTAGCCACCCGAGGAGCC-3’. The PCR was carried out by following program: 1 cycle at 94°C for 4 min, followed by 30 cycles at 94°C for 30 sec, annealing at 55 °C for 40 sec, and 72°C for 50 sec. These PCR products were sequencing by Mission Biotech company.

CyTOF mass cytometry

RBC lysis buffer (blood volume: lysis buffer volume = 1:1) was added to whole blood sample (with EDTA) at room temperature for 5 min to remove the red blood cells. Then a neutralization reaction with PBS (sample volume: PBS=1:1) and centrifugation at 300 g for 10 min was undertaken. The supernatant was removed and resuspended with 1mL of PBS and counted. The sample was prepared (3 x 10^6 cells/ 50 μL Maxpar Cell Staining Buffer) for the staining step as described in the protocol of Maxpar® Mouse Sp/LN Phenotyping Panel Kit (Fluidigm, South San Francisco, CA, USA) and performed using CyTOF® 2 Mass Cytometer (Fluidigm, South San Francisco CA, USA) at the Genomics Research Center, Academia Sinica). CyTOF data were uploaded and analyzed using Cytobank (Beckman Coulter Life Sciences, Pasadena, CA, USA), and gating strategy was applied to identify cell populations was used for making the viSNE plots. Cell populations were TCRb, CD3e, CD4, CD8a, B220, CD19, CD25, Gr-1, CD11b, CD11c, CD44, CD62L and NK1.1 **(Figure S5C)**.

CyTOF data preprocessing, clustering, and dimensionality reduction

We analyzed blood clusters of 25 thousand cells in Cytobank and set the parameters (Seed: Random, #Iterations: 1000, Perplexity: 30, Theta: 0.5). datasets are based on the following marker proteins **(Table 4)**: B cells ($CD45^+$ $B220^+$ $CD19^+$), CD3 T cells ($CD45^+$ $B220^-$ $CD19^-$ $CD3e^+$ $TCRb^+$), CD4 T Cells ($CD45^+$ $B220^-$ $CD19^-$ $CD3e^+$ $TCRb^+$ $CD4^+$), Naïve CD4 T Cells ($CD45^+$ $B220^-$ $CD19^-$ $CD3e^+$ $TCRb^+$ $CD4^+$ $CD44^-$ $CD62L^+$), Central Memory CD4 T Cells ($CD45^+$ $B220^-$ $CD19^-$ $CD3e^+$ $TCRb^+$ $CD4^+$ $CD44^+$ $CD62L^+$), Effector Memory CD4 T Cells ($CD45^+$ $B220^-$ $CD19^-$ $CD3e^+$ $TCRb^+$ $CD4^+$ $CD44^+$ $CD62L^-$), CD8 T cells ($CD45^+$ $B220^-$ $CD19^-$ $CD3e^+$ $TCRb^+$ $CD8a^+$), Naïve CD8 T Cells ($CD45^+$ $B220^-$ $CD19^-$ $CD3e^+$ $TCRb^+$ $CD8a^+$ $CD44^-$ $CD62L^+$), Central Memory CD8 T Cells ($CD45^+$ $B220^-$ $CD19^-$ $CD3e^+$ $TCRb^+$ $CD8a^+$ $CD44^+$

CD62L^{+}), Effector Memory CD8 T Cells (CD45^{+} B220^{-} CD19^{-} CD3e^{+} TCRb^{+} CD8a^{+} CD44^{+} CD62L^{-}), Regulatory T Cells (CD45^{+} B220^{-} CD19^{-} CD3e^{+} TCRb^{+} CD4^{+} CD25^{+}), Nature Killer T Cells (CD45^{+} B220^{-} CD19^{-} CD3e^{+} TCRb^{+} NK1.1^{+}), Natural Killer Cells (CD45^{+} B220^{-} CD19^{-} CD3e^{-} TCRb^{-} NK1.1^{+} CD11b^{-}), Dendritic Cells (CD45^{+} B220^{-} CD19^{-} CD3e^{-} TCRb^{-} NK1.1^{-} CD11b^{+} CD11c^{+}), Granulocytes (CD45^{+} B220^{-} CD19^{-} CD3e^{-} TCRb^{-} NK1.1^{-} CD11b^{+} CD11c^{-} Gr-1^{+}) and Monocytes/Macrophages (CD45^{+} B220^{-} CD19^{-} CD3e^{-} TCRb^{-} NK1.1^{-} CD11b^{+} CD11c^{-} Gr-1^{-}).

B16F10 Pulmonary Metastasis Assay

B16F10 (CRL6475™) cells were obtained from American Type Culture Collection (ATCC®) and cultured in Dulbecco’s Modified Eagle’s Medium (DMEM, Gibco, Thermo Fisher Scientific) with 10% fetal bovine serum (FBS, Gibco, Thermo Fisher Scientific) and 5% CO2 at 37°C. B16F10 cells were prepared and administered as described in Overwijk et al.,[6] 2×10^5 cells in 200 μL PBS (Gibco, Thermo Fisher Scientific) was injected into the tail vein of 8 weeks old mice. At 14 days post-injection of B16F10 cells, mice were euthanized, and the lungs were photographed for examination of the tumor nodules. Lung metastatic nodules were counted under a dissection microscope by investigators that were blinded to the treatment. A separate group of mice were challenged with B16F10 cells by an identical procedure and the survival time of mice was recorded after tail vein injection with B16F10 cells.

Subcutaneous tumor growth assay

B16F10, Lewis Lung Carcinoma, CRL-1642 (LLC) and T lymphoblast, TIB-39 (EL4)

were obtained from ATCC and cultured in DMEM (10% FBS) at 5% CO_2 at 37°C. Eight weeks old mice were injected subcutaneously with 2×10^5 cells in 200 μL PBS, and under observation at 7, 9, 11,13, and 15 days. Then, mice were euthanized, and the tumors were weighed.

Tumor-infiltrating immune cells assay

After the B16F10 pulmonary metastasis assay, mouse lungs were removed, fixed in paraffin, and sectioned into slices. The tissue slices were stained with anti-CD8a (Cat No. 14-0081-82; eBioscience, ThermoFisher) and anti-NK-1.1 (Cat No.108702; Biolegend, San Diego, CA, USA). CD8a$^+$ and NK1.1$^+$ cells were counted under the microscope which were infiltrating to the tumor (T) and NAT (not a tumor). The value was calculated as T/NAT (cells/μm^2).

Flow cytometric analysis of splenic immune cells

Splenocytes were isolated from the spleen of each *Klf1*$^{+/+}$ and *Klf1*$^{K74R/K74R}$ mice bearing B16F10 mouse melanoma. Single-cell suspensions were prepared by mincing the spleen through a 70 and 40 μm cell strainer (Falcon, BD Biosciences, Becton, Singapore). Splenic red blood cells were removed by RBC lysis buffer (Viogene, New Taipei City, Taiwan) and following further centrifugation, lymphocytes were re-suspended with staining buffer (autoMACS Running Buffer, Miltenyi Biotec, Bergisch Gladbach, Germany). Approximately 1×10^7 cells were processed for flow cytometry using the following marker of NK cells (CD3e$^-$, NK1.1$^+$), CD8$^+$ T cells (CD3e$^+$, CD8a$^+$) and CD8$^+$ Effector Cells (CD3e$^+$, CD8a$^+$, CD44$^+$, CD62L$^-$) were from BD Pharmingen or BD Horizon (BD Biosciences).

Hematopoietic stem cell infusion (HSCI)

Three months old, young donor *Klf1*$^{+/+}$and *Klf1*$^{K74R/K74R}$ mice were euthanized, and bone marrow was extracted, respectively. The hematopoietic stem cells (Lin^{-}, cKit^{+}, Sca1^{+}) were isolated by the Lineage Cell Depletion Kit (Miltenyi Biotec). The 1 x 10^{6} hematopoietic stem cells in 200 μL PBS were injected into the tail vein of 25.5 months old *Klf1*$^{+/+}$ mice. The appearance and survival time of mice were recorded after tail vein injection with HSCs. The HSCI was once every 2 weeks for 8 cycles (about 4 months).

Supplementary figure legends

Supplementary figures

Figure S1

(A)

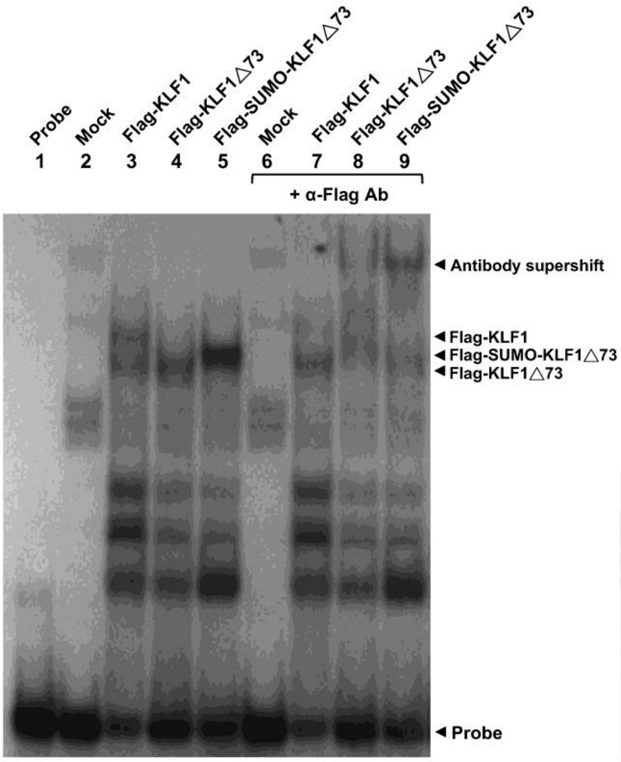

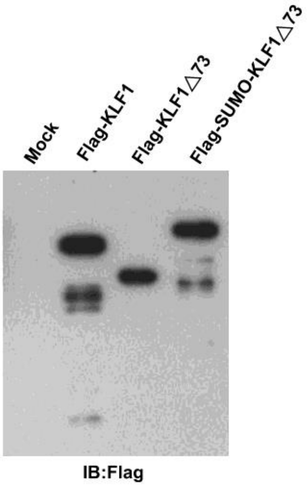

Figure S1

(B) Loss-of-native-allele assay

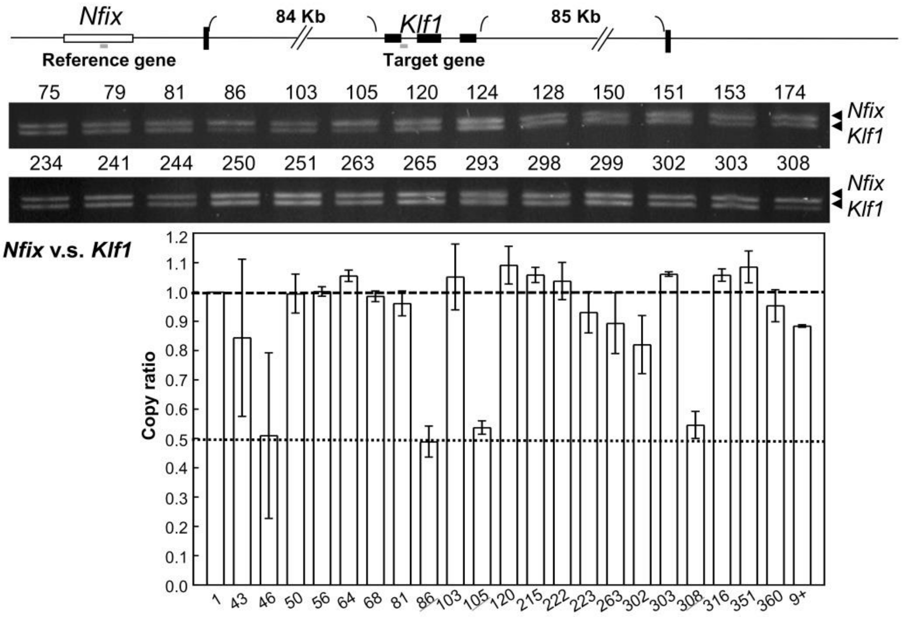

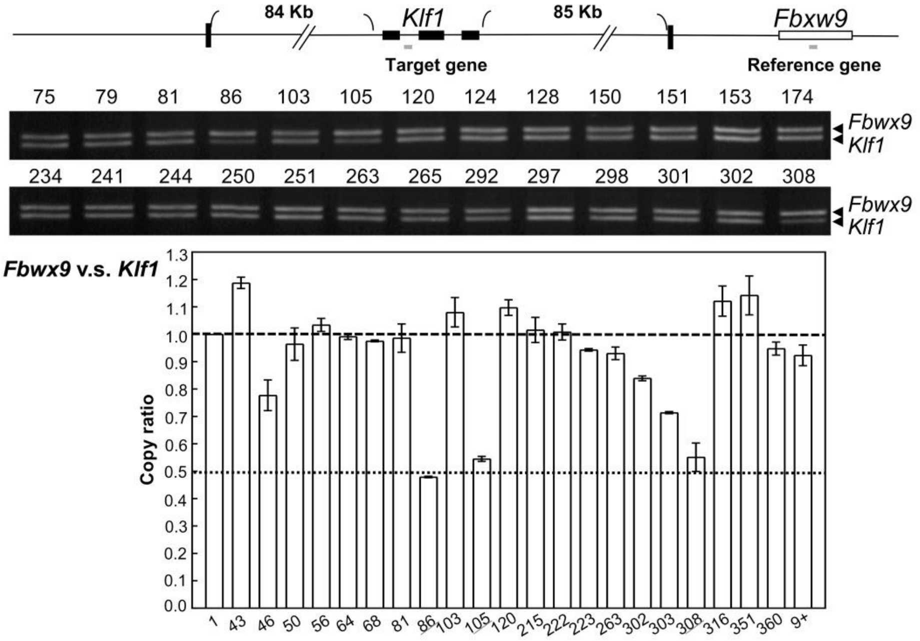

Figure S1

(C)

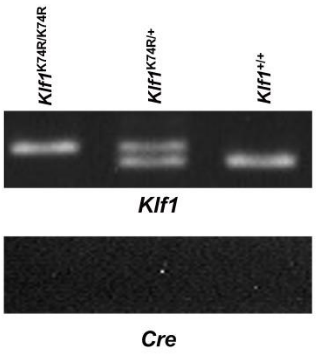

(D)

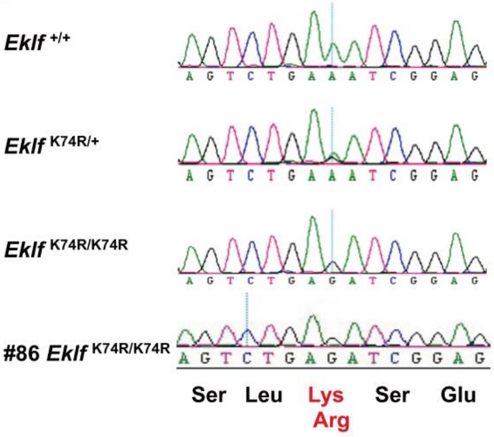

(E)

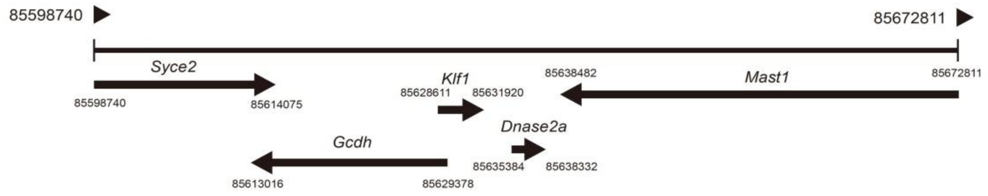

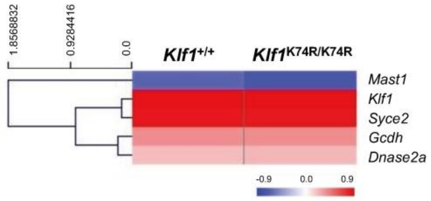

	Klf1$^{+/+}$	*Klf1*$^{K74R/K74R}$	*Klf1*$^{K74R/K74R}$ / *Klf1*$^{+/+}$	
Gene name	Mean±SD		Effect size	*P*
Gcdh	8.3803±0.2947	8.4884±0.094	0.1081	0.5971
Dnase2a	7.4986±0.5446	7.6561±0.3998	0.1575	0.7088
Mast1	6.4939±0.3252	6.8335±0.1078	0.3396	0.2053
Syce2	11.6398±0.2456	11.659±0.1722	0.0192	0.9178
Klf1	12.4249±0.4549	12.8389±0.1661	0.4140	0.2514

Figure S1

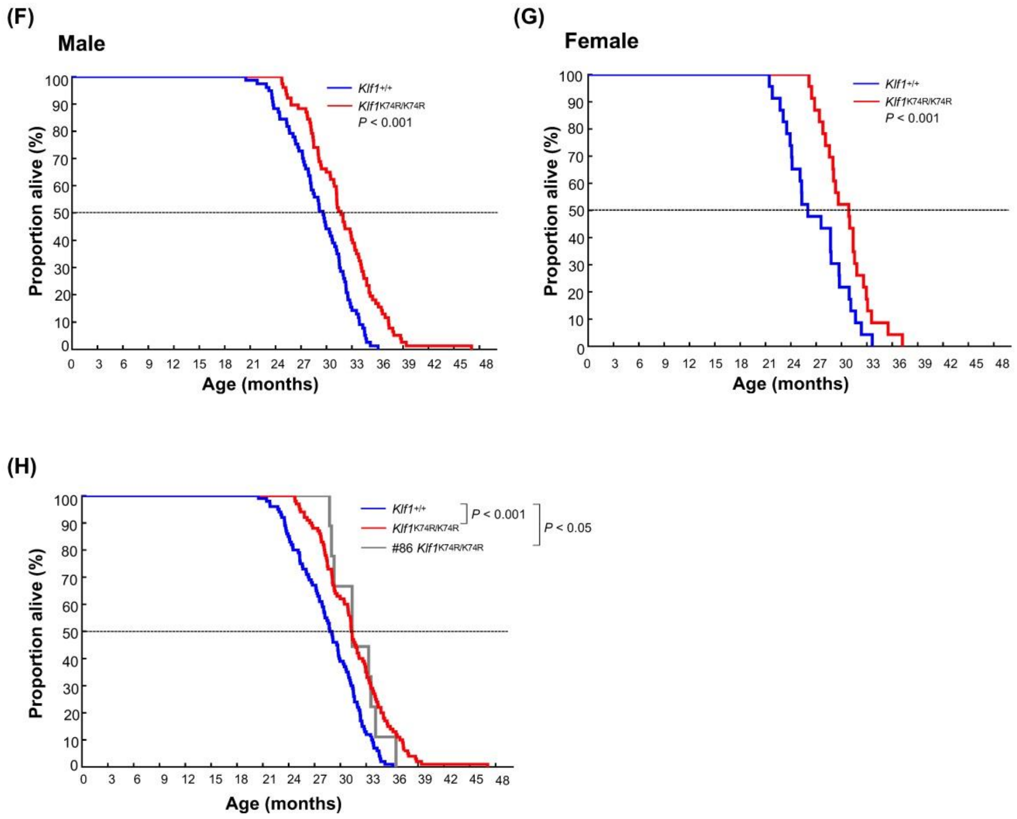

Figure S1. Generation of *Klf1*$^{\mathbf{K74R/K74R}}$ knockin mouse model.

(A) Effect of SUMOylation on DNA binding activity *in vitro*. Left panel, EMSA was performed using cell extracts obtained from HEK293T transiently expressing mock control (lanes 2 and 6), Flag-KLF1 (lanes 3 and 7), Flag-KLF1△73 (lanes 4 and 8), and Flag-SUMO-KLF1△73 (lanes 5 and 9), respectively, incubated with ^{32}P-labeled

probe containing CACCC box from the β-major globin promoter (lane 1) and then subjected into 6% PAGE. For supershift assay (lanes 6 to 9), anti-Flag antibodies were added into reaction mixtures followed by incubation with the P-labeled probe. The positions of various KLF1-DNA binding complex and supershift bands are denoted by the arrowheads. An equal amount of Flag-KLF1, Flag-KLF1△73, and Flag-SUMO-KLF1△73 containing cell extracts were analyzed by anti-Flag immunoblotting, as shown in the right panel, and then applied to EMSA. **(B)** 5FA/P5RA/5RA was used as a primer to screen 375 ES cell colonies via PCR. The copy number of the native *Klf1* gene was screened in 375 ES cell colonies via loss-of-native-allele assay (Valenzuela et al.,2003). The dPCR (digital PCR instrument) was standardized to two nearby reference genes, namely *Nfix* and *Fbwx9*. The primer list is included in the Supplementary Materials. The integrity of the three gene loci used in dPCR was preliminarily verified via PCR. Loss of a copy of the native *Klf1* gene indicated successful gene targeting. The target gene is reduced from two copies to one copy in the genome, in contrast to the reference gene (*Nfix*, upper panel or *Fbwx9*, lower panel). Two primary screens of 375 ES cell colonies, in each genome and with respect to the genetic alteration, can be unequivocally distinguished by two parallel locus quantification, in each of which a single uPCR or dPCR probe is compared to a single reference gene to yield a ΔCT value. ES cell colonies (375) were screened by PCR analysis of their genomic DNAs using PCR primer, gt *Klf1* 3'/gt *Klf1* d 5'/gt *Klf1* PGK5'. **(C)** Genotyping of tail DNAs was conducted to identify WT (*Klf1*$^{+/+}$), heterozygous (*Klf1*$^{K74R/+}$), and homozygous (*Klf1*$^{K74R/K74R}$) mice. Mouse tail genomic DNAs were analyzed by PCR using appropriate primers. **(D)** Examples of cDNA sequencing analysis of the *Klf1* exon2 region. The lysine (K) at amino acid position 74 of *Klf1* was replaced by arginine (R) due to a change of AAA to AGA. **(E)**

The heatmap shows the expression of four genes nearby *Klf1*: *Mast1*, *Syce2*, *Gcdh* and *Dnase2a*. After normalization with MAS5 algorithm, All expression values were divided by the median and converted to log2 values. The blue color represents a lower and the red color represents a higher expression level. Survival analysis of male (n = 77) **(E)** and female (n=23) **(F)** *Klf1*$^{+/+}$and *Klf1*$^{K74R/K74R}$ mice. Each data point represents one animal (n, number of animals in each cohort). The different parameters are listed in **Table 1**. (G) Kaplan-Meier survival curves of the *Klf1*$^{+/+}$, *Klf1*$^{K74R/K74R}$ (n = 100 mice for each genotype) and #86 *Klf1*$^{K74R/K74R}$ (n = 9) mice. Each data point represents one animal. Statistical significance was assessed using the original method of the log-rank test (*Klf1*$^{+/+}$ vs. *Klf1*$^{K74R/K74R}$ and *Klf1*$^{+/+}$ vs. #86 *Klf1*$^{K74R/K74R}$).

Figure S2

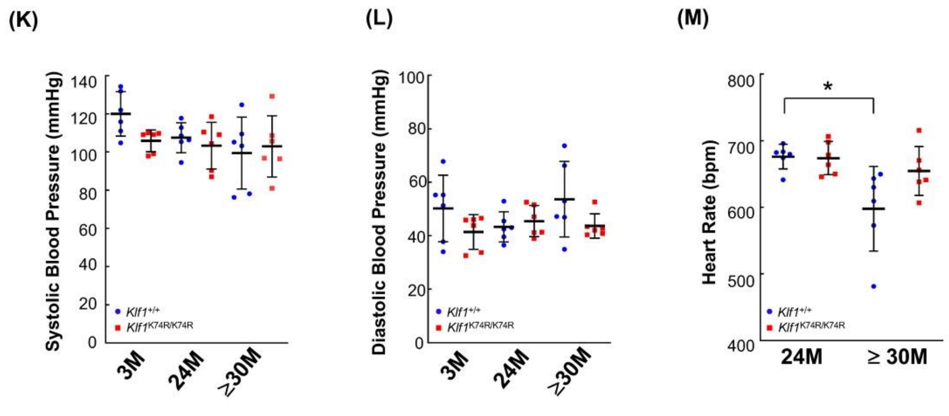

(K)
Systolic Blood Pressure (mmHg)
140
120
100
80
60
40
20
0
Klf1+/+
Klf1K74R/K74R
3M
24M
≥30M
(L)
Diastolic Blood Pressure (mmHg)
100
80
60
40
20
0
Klf1+/+
Klf1K74R/K74R
3M
24M
≥30M
(M)
Heart Rate (bpm)
800
700
600
500
400
*
Klf1+/+
Klf1K74R/K74R
24M
≥ 30M

Figure S2

(A)

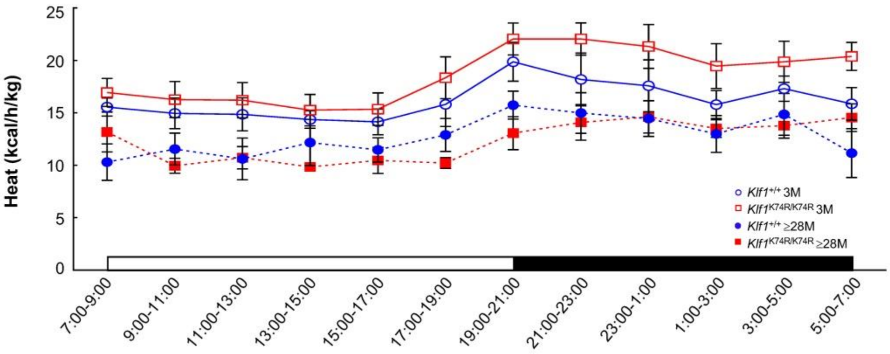

(B)

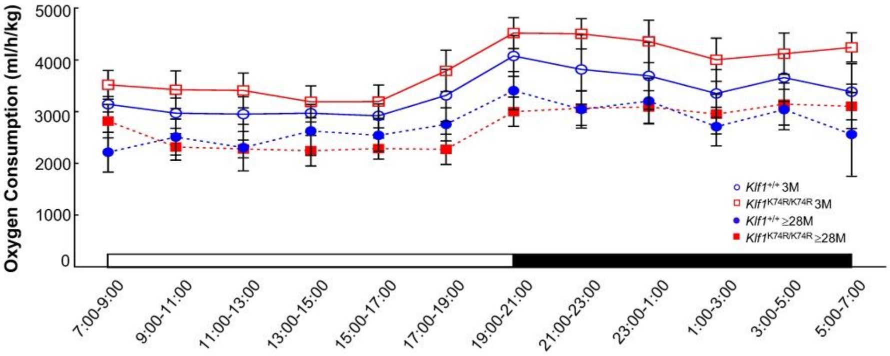

(C)

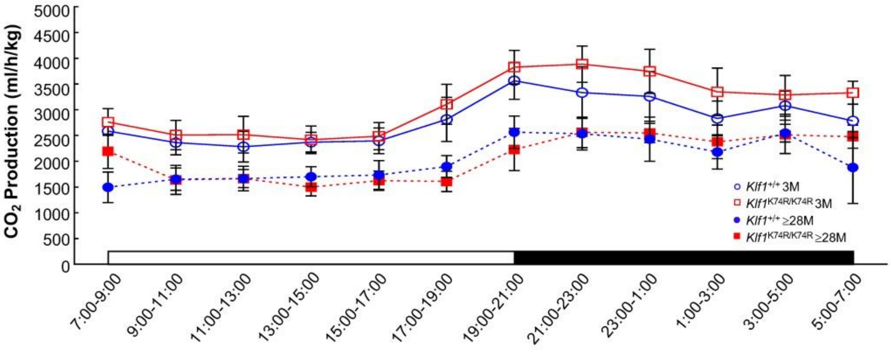

Figure S2

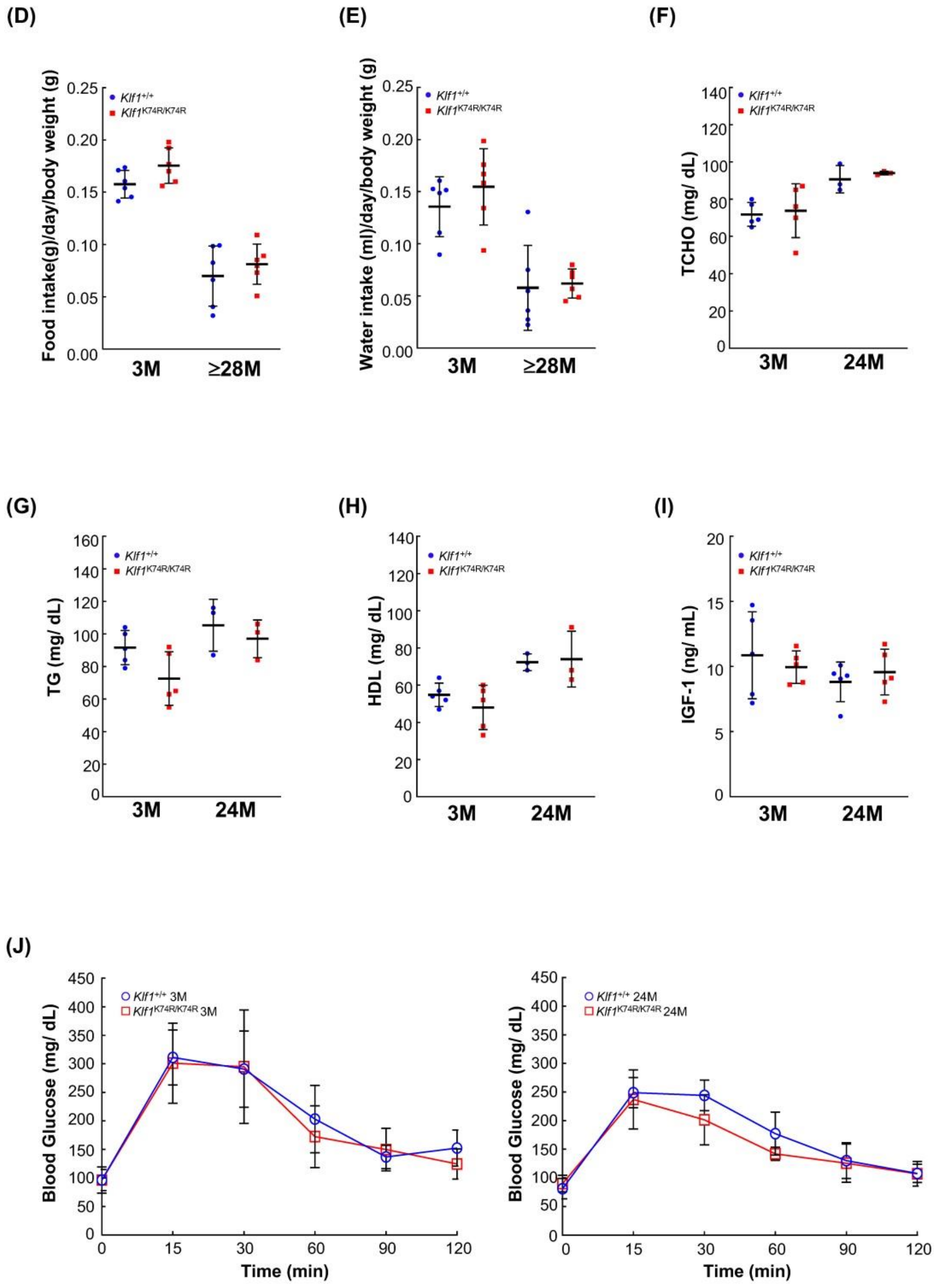

Figure S2. Physiological performance of *Klf1*$^{K74R/K74R}$ mice. The canonical diurnal metabolic parameters in young (3 months old) and old (≥ 28 months old) *Klf1*$^{+/+}$ and *Klf1*$^{K74R/K74R}$ mice were measured and compared, including (A) heat, (B) oxygen consumption, and (C) CO_2 production (n = 6, males, respectively). (D) Food intake parameters are compared with *Klf1*$^{+/+}$ and *Klf1*$^{K74R/K74R}$ mice, respectively (n = 6,

males, respectively). (E) Water intake parameters are compared with *Klf1*$^{+/+}$ and *Klf1*$^{K74R/K74R}$ mice, respectively (n = 6, males). Level of (F) total cholesterol (TCHO), (G) triglyceride (TG), (H) high-density lipoprotein (HDL) of blood samples from 3 months (n = 5, males) and 24 months old (n = 3, males) *Klf1*$^{+/+}$ and *Klf1*$^{K74R/K74R}$ mice were analyzed and compared. (I) Insulin-like growth factor I (IGF-1) of blood samples from 3 months and 24 months old *Klf1*$^{+/+}$ and *Klf1*$^{K74R/K74R}$ mice (n = 5, males, respectively) were analyzed and compared. Blood levels of (J) glucose tolerance was also compared of 3 months (n = 7, left panel, males), 24 months (n = 6, right panel, males). Presented are the comparisons of (K) systolic blood pressure, (L) diastolic blood pressure, and (M) heart rate of 24 months and over 30 months old *Klf1*$^{+/+}$ and *Klf1*$^{K74R/K74R}$ mice (n = 5, males, respectively) were analyzed and compared. Statistical significance was assessed by student's t-test, $*P < 0.05$ as compared with mice of different ages.

Figure S3

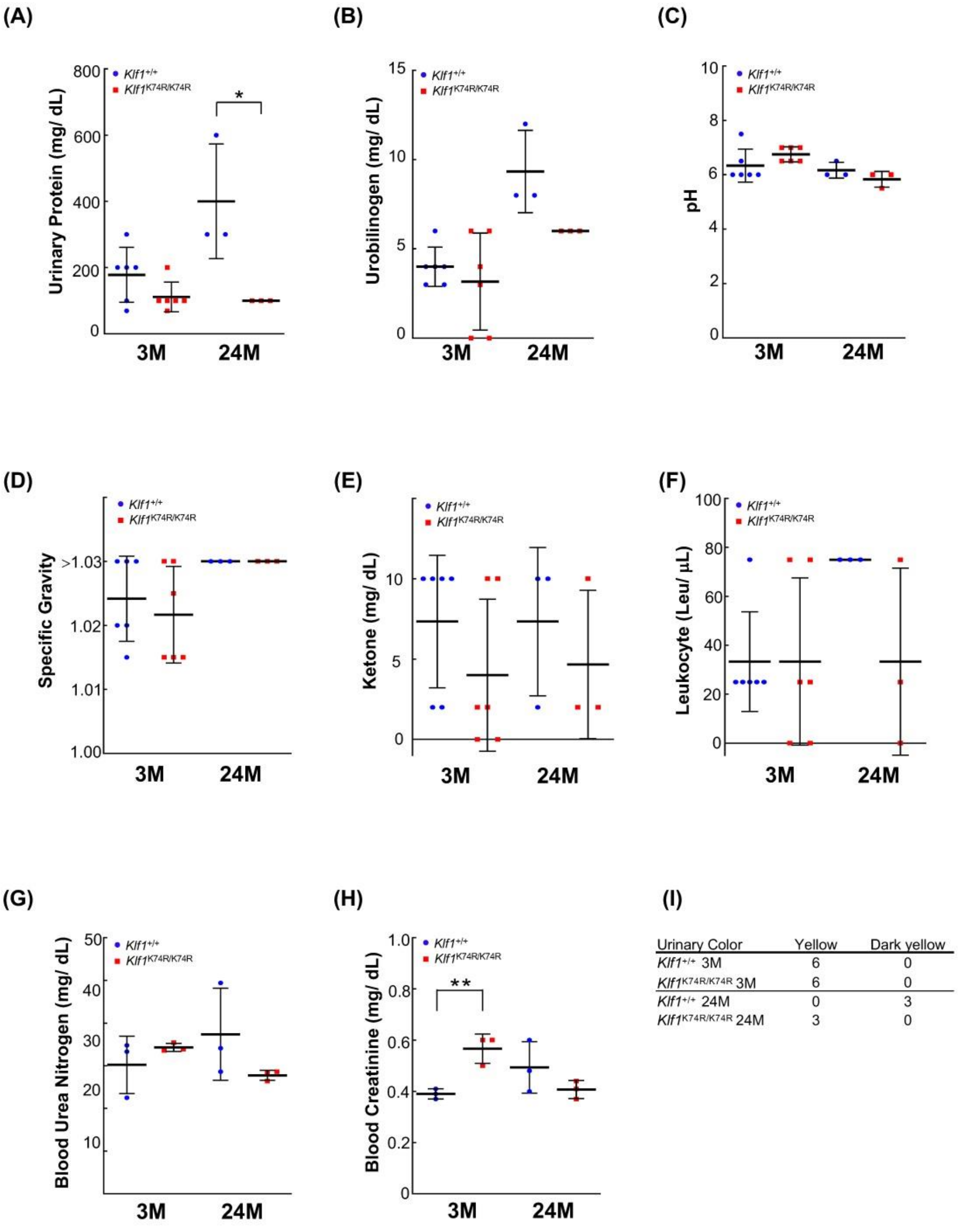

Urinary Color	Yellow	Dark yellow
Klf1+/+ 3M	6	0
*Klf1*K74R/K74R 3M	6	0
Klf1+/+ 24M	0	3
*Klf1*K74R/K74R 24M	3	0

Figure S3. Urine physiology assay of *Klf1*$^{K74R/K74R}$ mice.

The urinalysis of (A) protein (B) urobilinogen, (C) pH value, (D) specific gravity, (E) ketone, and (F) leukocytes of 3 months (n = 6, males) and 24 months old (n = 3, males) *Klf1*$^{+/+}$ and *Klf1*$^{74R/K74R}$ mice were measured and compared. Blood chemistry of (G) blood urea nitrogen (BUN) and (H) blood creatinine was also be measured in 3

months (n = 3, males) and 24 months old (n = 3, males) *Klf1*$^{+/+}$ and *Klf1*$^{K74R/K74R}$ mice. (I) The urinary color of 3 months (n = 6, males) and 24 months old (n = 3, males) *Klf1*$^{+/+}$ and *Klf1*$^{K74R/K74R}$ mice were observed. Statistical significance was assessed by student's t-test, *$P < 0.05$, **$P < 0.01$ as compared with mice of different genome types.

Figure S4

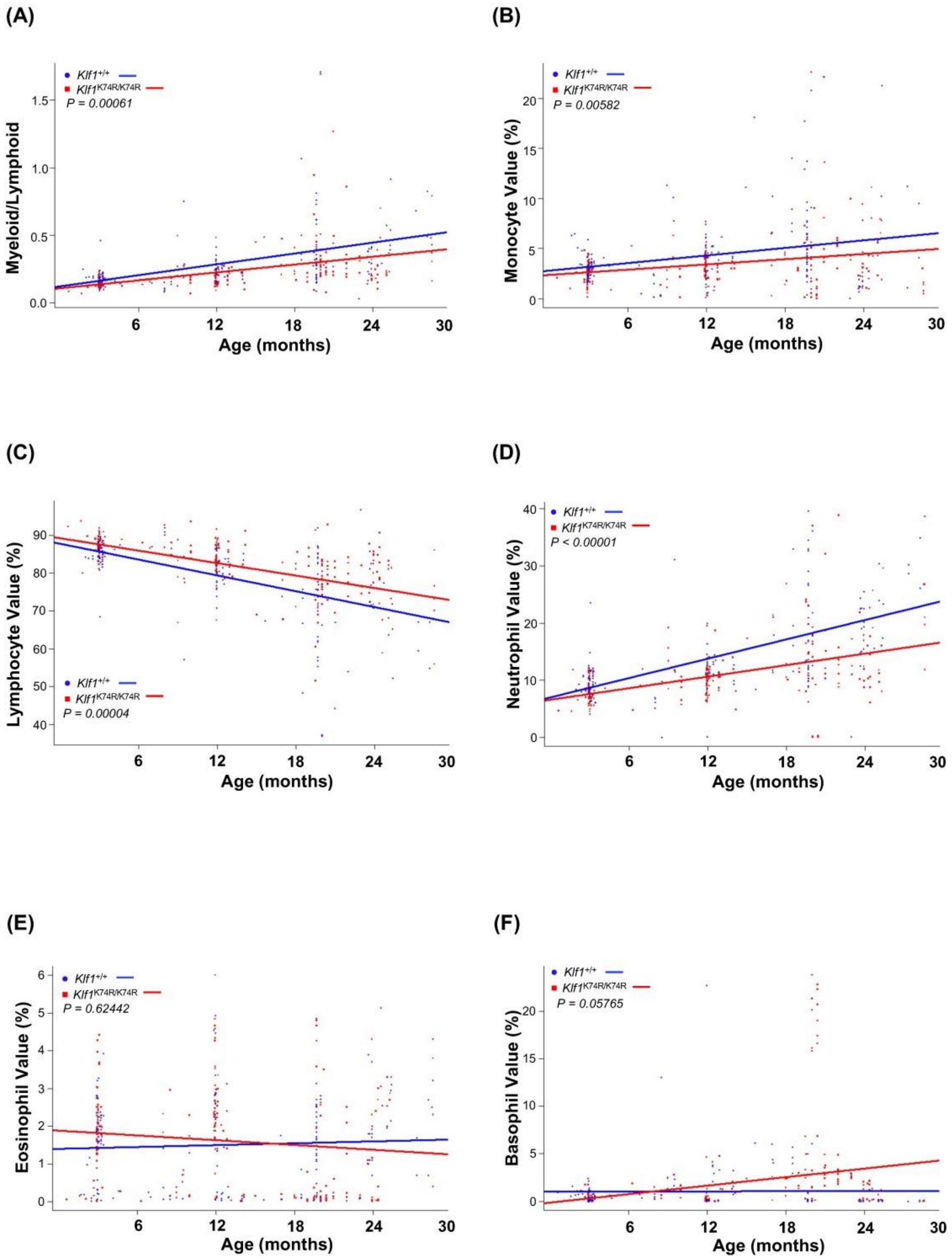
(A)
Klf1+/+
Klf1K74R/K74R
P = 0.00061
Myeloid/Lymphoid
Age (months)
(B)
Klf1+/+
Klf1K74R/K74R
P = 0.00582
Monocyte Value (%)
Age (months)
(C)
Klf1+/+
Klf1K74R/K74R
P = 0.00004
Lymphocyte Value (%)
Age (months)
(D)
Klf1+/+
Klf1K74R/K74R
P < 0.00001
Neutrophil Value (%)
Age (months)
(E)
Klf1+/+
Klf1K74R/K74R
P = 0.62442
Eosinophil Value (%)
Age (months)
(F)
Klf1+/+
Klf1K74R/K74R
P = 0.05765
Basophil Value (%)
Age (months)

Figure S4

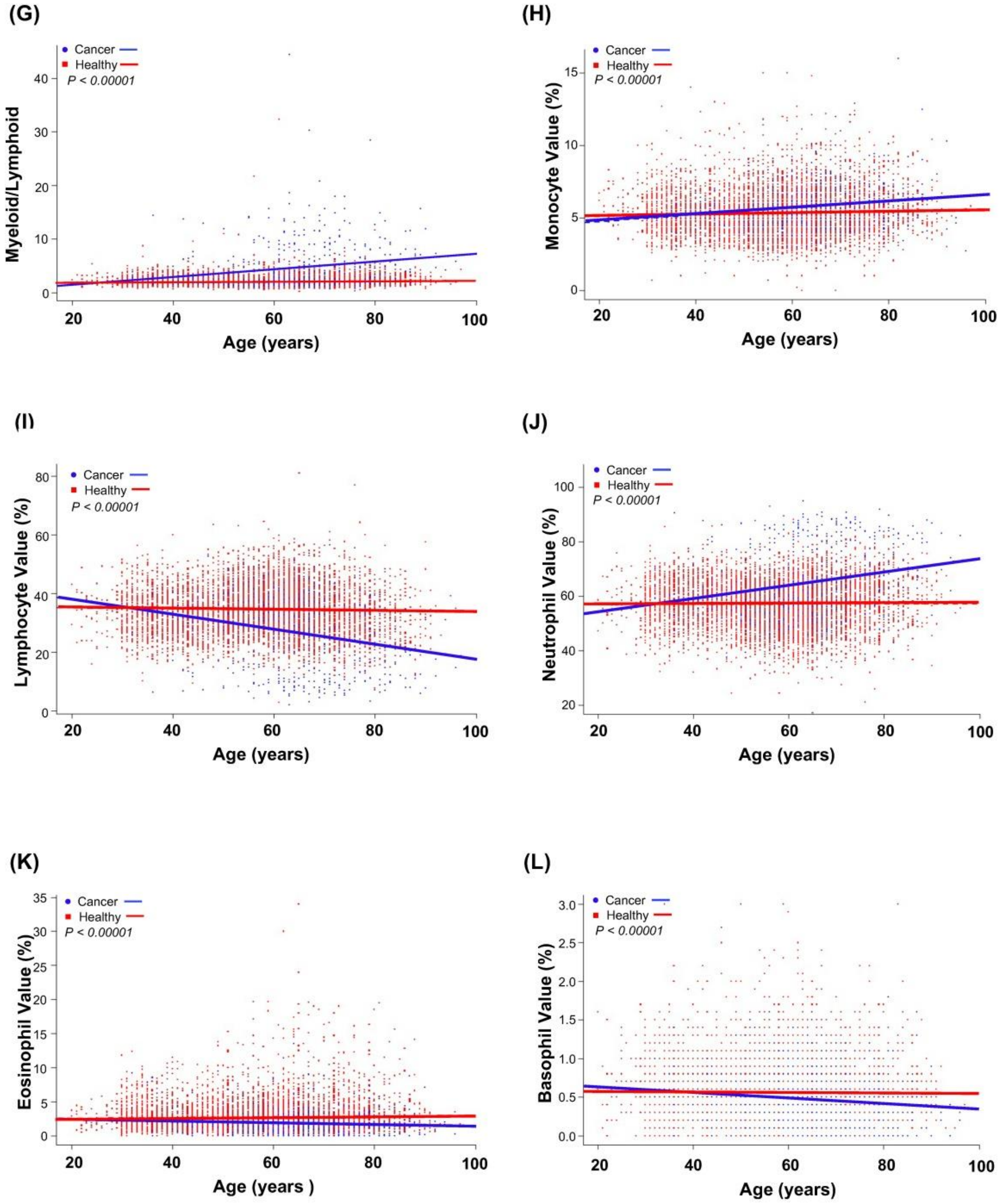

Figure S4. The hematopoietic cell sub-population of *Klf1*$^{K74R/K74R}$ mice comparison to *Klf1*$^{+/+}$ and healthy comparison to cancer populace.

(A) (G) The inflammatory biomarkers of mice (n = 369) and humans (n = 6637) were calculated including the myeloid-lymphoid ratio. The percentages of different cell sub-populations included (B) (H) monocyte value, (C) (I) lymphocyte value, (D) (J)

neutrophil value, (E) (K) eosinophil value, and (F) (L) basophil value in the peripheral blood of different ages mice and humans were assayed by complete blood count (CBC). Cells from peripheral blood, including of each age, were analyzed and compared. Statistical significance was assessed by ANCOVA. *$P < 0.05$, **$P < 0.01$, ***$P < 0.005$ as compared with mice of different genome types (*Klf1*$^{+/+}$ mice, n = 136; *Klf1*$^{K74R/K74R}$ mice, n = 233), and people's pathological state (HCC, n = 451; healthy, n = 6186).

Figure S5

(A)

LLC *S.C.*

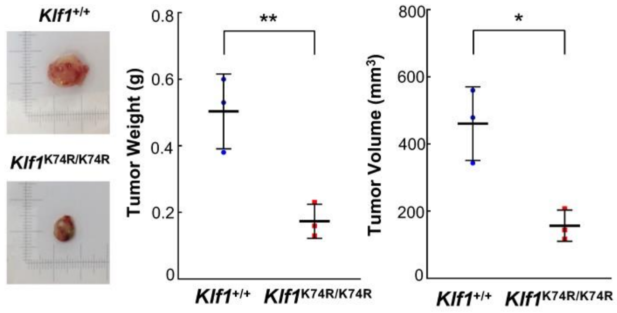

(B)

EL4 *S.C.*

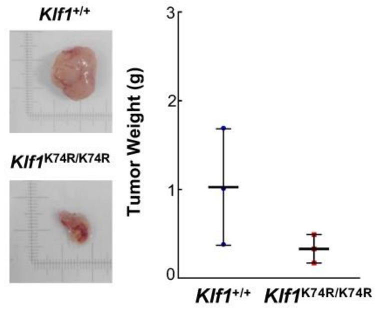

Figure S5

(C)

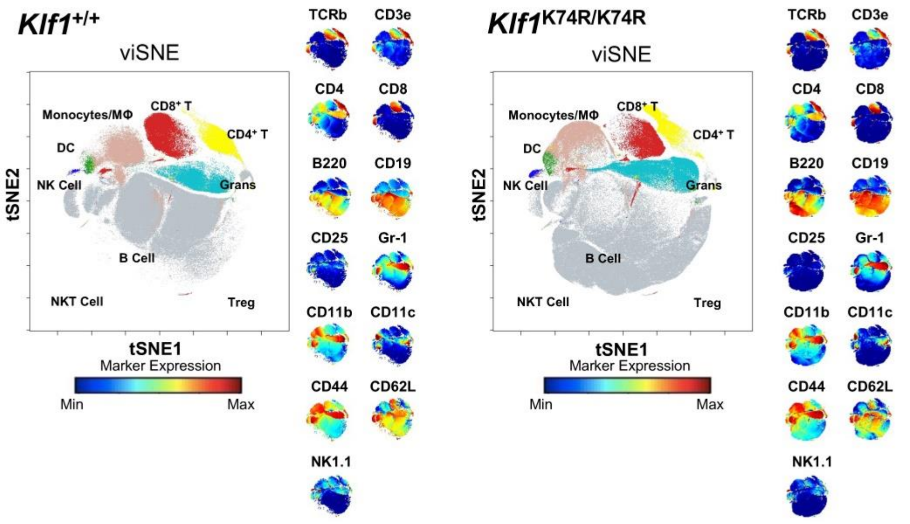
Klf1+/+
viSNE
Monocytes/MΦ
CD8+ T
CD4+ T
DC
NK Cell
Grans
B Cell
NKT Cell
Treg
tSNE2
tSNE1
Marker Expression
Min
Max
TCRb
CD3e
CD4
CD8
B220
CD19
CD25
Gr-1
CD11b
CD11c
CD44
CD62L
NK1.1
Klf1K74R/K74R
viSNE

(D)

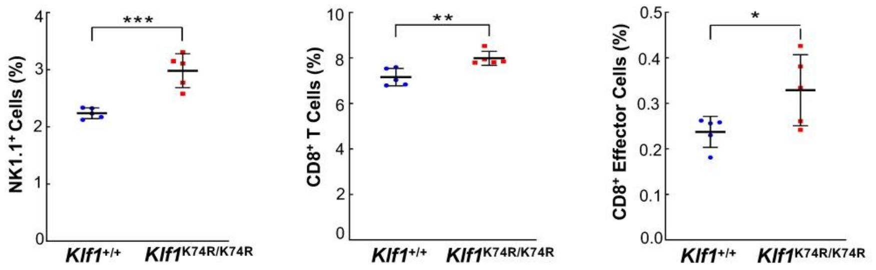
NK1.1+ Cells (%)
***
CD8+ T Cells (%)
**
CD8+ Effector Cells (%)
*
Klf1+/+
Klf1K74R/K74R

Figure S5

(E)

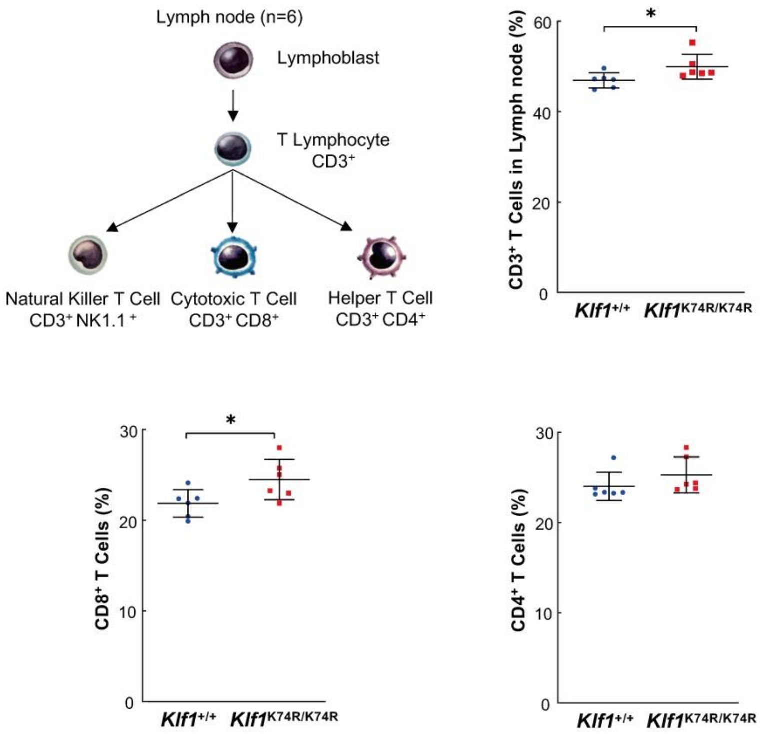

Figure S5. Anti-tumor effect of *Klf1*$^{K74R/K74R}$ mice.

The blood of 24 months old mice was assayed by CyTOF (n = 5, males). (A) Lewis lung carcinoma cells (LLC) and (B) mouse lymphoma cells (EL4) (2 x 10^{5} cells) were injected subcutaneously into 8 weeks old *Klf1*$^{+/+}$ (n = 3) or *Klf1*$^{K74R/K74R}$ (n = 3) mice, respectively. After two weeks, tumors were excised from each mouse. Tumor weight

and volume were measured and represented in the form of dots pairs. Statistical significance was assessed by student's t-test, * P < 0.05, ** P < 0.01 as compared with *Klf1*$^{+/+}$ and *Klf1*$^{K74R/K74R}$ mice. (C) The viSNE plots TCRb, CD3e, CD4, CD8a, B220, CD19, CD25, Gr-1, CD11b, CD11c, CD44, CD62L and NK1.1 presentations were drawn by Cytobank. (D) The mice were injected intravenously with 2 x 10^5 B16F10 melanoma cells, respectively (n = 5, males). Spleens were collected 14 days after tumor challenge and the nodules were counted. (E) The total lymphocytes ($CD3^+$ T cells) were harvested from lymph nodes of 3 months old *Klf1*$^{+/+}$ and *Klf1*$^{K74R/K74R}$ mice and analyzed by flow cytometry with use of appropriate antibodies including anti-CD3, anti-CD4, and anti-CD8. The splenocytes were isolated and assay by flow cytometry. All the data are presented as mean ± SD Statistical significance was assessed by student's t-test. *P < 0.05, **P < 0.01, and ***P < 0.005.

Supplementary Table

Table S1. Body composition of *Klf1*$^{+/+}$ and *Klf1*$^{K74R/K74R}$ mice.

Items	3 Months		24 Months		28 Months	
	Klf1$^{+/+}$ (n=7)	*Klf1*$^{K74R/K74R}$ (n=7)	*Klf1*$^{+/+}$ (n=7)	*Klf1*$^{K74R/K74R}$ (n=7)	*Klf1*$^{+/+}$ (n=3)	*Klf1*$^{K74R/K74R}$ (n=3)
Fat Mass (%)	8.82 ± 0.97	7.23 ± 0.88 *	8.16 ± 1.31	8.84 ± 0.85	10.07 ± 1.50	6.22 ± 0.50*
Lean Mass (%)	76.90 ± 1.30	78.17 ± 0.84	75.76 ± 0.90	76.48 ± 1.39	73.95 ± 0.65	76.53 ± 0.47*

* $P \leq 0.05$

Table S2. Complete blood count (CBC) data of *Klf1*$^{+/+}$ and *Klf1*$^{K74R/K74R}$ mice.

Item	3 Months			24 Months		
	Klf1$^{+/+}$ (n=40)	*Klf1*$^{K74R/K74R}$ (n=40)	*P*	*Klf1*$^{+/+}$ (n=18)	*Klf1*$^{K74R/K74R}$ (n=18)	*P*
WBC count (10^3/uL)	11.23 ± 2.30	10.51 ± 1.97	0.14	11.96 ± 3.26	8.62 ± 3.06	0.00
RBC count (10^6/uL)	11.41 ± 0.50	11.12 ± 0.56	0.02	10.18 ± 0.65	9.96 ± 0.64	0.33
HGB (g/dL)	16.75 ± 0.72	16.57 ± 0.70	0.27	14.74 ± 0.82	14.51 ± 1.01	0.47
HCT (%)	52.93 ± 2.37	51.69 ± 2.07	0.02	49.35 ± 2.76	46.86 ± 3.17	0.02
MCV (fL)	46.43 ± 1.57	46.51 ± 1.27	0.81	48.48 ± 1.36	47.05 ± 1.76	0.01

MCH (pg)	14.68 ± 0.26	14.90 ± 0.36	0.00	14.48 ± 0.40	14.57 ± 0.38	0.54
MCHC (g/dL)	31.66 ± 1.27	32.07 ± 0.94	0.12	29.88 ± 0.78	30.97 ± 0.94	0.00
PLT count (10^3/uL)	1366.25 ± 166.19	1268.05 ± 177.74	0.01	2105.06 ± 382.32	2073.22 ± 713.42	0.88
RDW-CV (%)	21.65 ± 1.12	21.20 ± 0.58	0.03	22.87 ± 1.74	21.18 ± 1.36	0.00
Neutrophils count (10^3/uL)	1.03 ± 0.32	0.84 ± 0.23	0.00	1.78 ± 0.68	1.23 ± 0.41	0.01
Lymphocyte count (10^3/uL)	9.65 ± 1.99	9.14 ± 1.81	0.24	9.53 ± 2.62	6.67 ± 2.63	0.00
Monocyte count (10^3/uL)	0.29 ± 0.13	0.28 ± 0.09	0.78	0.29 ± 0.15	0.47 ± 0.24	0.02
Eosinophils count (10^3/uL)	0.25 ± 0.11	0.24 ± 0.09	0.54	0.35 ± 0.29	0.25 ± 0.08	0.18
Basophils count (10^3/uL)	0.02 ± 0.02	0.02 ± 0.02	0.94	0.01 ± 0.02	0.02 ± 0.03	0.33
Neutrophils (%)	9.16 ± 2.07	8.10 ± 2.02	0.03	14.68 ± 3.56	15.00 ± 4.30	0.82
Lymphocyte (%)	85.87 ± 2.30	86.80 ± 2.40	0.09	79.80 ± 3.91	76.34 ± 5.34	0.04
Monocyte (%)	2.59 ± 0.98	2.67 ± 0.75	0.69	2.69 ± 1.56	5.49 ± 2.29	0.00
Eosinophils (%)	2.23 ± 0.67	2.28 ± 0.79	0.76	2.75 ± 1.72	3.02 ± 0.97	0.58
Basophils (%)	0.16 ± 0.15	0.16 ± 0.16	0.98	0.09 ± 0.21	0.15 ± 0.30	0.52

Reference

[1] J. J. Liu, S. C. Hou, C. K. Shen, *J Biol Chem* **2003**, *278* (21), 19534, https://doi.org/10.1074/jbc.M212278200.

[2] Y. C. Shyu, S. C. Wen, T. L. Lee, X. Chen, C. T. Hsu, H. Chen, R. L. Chen, J. L. Hwang, C. K. Shen, *Cell Res* **2006**, *16* (4), 347, https://doi.org/10.1038/sj.cr.7310045.

[3] a) T. L. Lee, Y. C. Shyu, P. H. Hsu, C. W. Chang, S. C. Wen, W. Y. Hsiao, M. D. Tsai, C. K. Shen, *Proc Natl Acad Sci U S A* **2010**, *107* (1), 52, https://doi.org/10.1073/pnas.0909153107; b) Y. C. Shyu, T. L. Lee, C. Y. Ting, S. C. Wen, L. J. Hsieh, Y. C. Li, J. L. Hwang, C. C. Lin, C. K. Shen, *Mol Cell Biol* **2005**, *25* (23), 10365, https://doi.org/10.1128/MCB.25.23.10365-10378.2005.

[4] J. B. Weir, *J Physiol* **1949**, *109* (1-2), 1,

https://doi.org/10.1113/jphysiol.1949.sp004363.

[5] C. Knoess, S. Siegel, A. Smith, D. Newport, N. Richerzhagen, A. Winkeler, A. Jacobs, R. N. Goble, R. Graf, K. Wienhard, W. D. Heiss, *Eur J Nucl Med Mol Imaging* **2003**, *30* (5), 737, https://doi.org/10.1007/s00259-002-1052-6.

[6] W. W. Overwijk, N. P. Restifo, *Curr Protoc Immunol* **2001**, *Chapter 20*, Unit 20.1, https://doi.org/10.1002/0471142735.im2001s39.
